# Vaginal microbial community state types fail to predict IVF outcomes, whereas *Ureaplasma parvum* and *Lactobacillus iners* are negative predictors of implantation, clinical pregnancy, and live birth

**DOI:** 10.1093/hropen/hoag018

**Published:** 2026-03-05

**Authors:** Simon Graspeuntner, Mariia Lupatsii, Noemi Hamala, Antonia Masuch, Marion Depenbusch, Iris Pfeffer, Askan Schultze-Mosgau, Tanja K Eggersmann, Jan Rupp, Georg Griesinger

**Affiliations:** Institute of Medical Microbiology, University of Lübeck, Lübeck, Germany; German Centre for Infection Research, Partner Site Hamburg-Lübeck-Borstel-Riems, Lübeck, Germany; Medical Clinic III, University Hospital Schleswig-Holstein, Lübeck, Germany; Institute of Medical Microbiology, University of Lübeck, Lübeck, Germany; Department of Gynaecological Endocrinology and Reproductive Medicine, University Hospital of Schleswig-Holstein, Campus Lübeck and Universitäres Kinderwunschzentrum Lübeck und Manhagen, Lübeck, Germany; Department of Gynaecological Endocrinology and Reproductive Medicine, University Hospital of Schleswig-Holstein, Campus Lübeck and Universitäres Kinderwunschzentrum Lübeck und Manhagen, Lübeck, Germany; Department of Gynaecological Endocrinology and Reproductive Medicine, University Hospital of Schleswig-Holstein, Campus Lübeck and Universitäres Kinderwunschzentrum Lübeck und Manhagen, Lübeck, Germany; Institute of Medical Microbiology, University of Lübeck, Lübeck, Germany; Department of Gynaecological Endocrinology and Reproductive Medicine, University Hospital of Schleswig-Holstein, Campus Lübeck and Universitäres Kinderwunschzentrum Lübeck und Manhagen, Lübeck, Germany; Department of Gynaecological Endocrinology and Reproductive Medicine, University Hospital of Schleswig-Holstein, Campus Lübeck and Universitäres Kinderwunschzentrum Lübeck und Manhagen, Lübeck, Germany; Institute of Medical Microbiology, University of Lübeck, Lübeck, Germany; German Centre for Infection Research, Partner Site Hamburg-Lübeck-Borstel-Riems, Lübeck, Germany; Infectious Disease Clinic, University Hospital Schleswig-Holstein, Lübeck, Germany; Department of Gynaecological Endocrinology and Reproductive Medicine, University Hospital of Schleswig-Holstein, Campus Lübeck and Universitäres Kinderwunschzentrum Lübeck und Manhagen, Lübeck, Germany

**Keywords:** IVF, assisted reproduction, vaginal microbiota, treatment prediction, *Ureaplasma parvum*, *Lactobacillus iners*, *Lactobacillus fornicalis*, dydrogesterone

## Abstract

**STUDY QUESTION:**

Are previously proposed vaginal microbial community state types (CSTs) valid predictors of IVF success, or do alternative microbial signatures provide stronger associations?

**SUMMARY ANSWER:**

Previously proposed CSTs as predictors of implantation, clinical pregnancy, and live birth were not confirmed, while an interaction between *Ureaplasma parvum* and *Lactobacillus iners* emerged as a strong negative predictor.

**WHAT IS KNOWN ALREADY:**

Infertility affects 17% of the global population. Only one-third of treatment cycles of assisted reproductive technologies result in embryo implantation, and even fewer lead to clinical pregnancy or live births. While early findings have spurred the development of microbiome-based tests for success prediction, evidence on supporting their reliability remains inconclusive.

**STUDY DESIGN, SIZE, DURATION:**

This prospective, single-centre study aimed to validate existing, and identify better, microbial predictors of infertility treatment outcomes. A cohort of 266 infertile female patients (age 18–45 years) undergoing a frozen-thawed embryo transfer cycle in an anovulatory regimen (i.e. a cycle with transfer of an embryo following a previous oocyte retrieval, fertilization, and freezing of embryos) was recruited for the study within a timeframe from May 2017 to March 2019.

**PARTICIPANTS/MATERIALS, SETTING, METHODS:**

The female, infertile patients, aged 18–45 years, were undergoing routine care. Vaginal swabs were taken prior to embryo transfer and subjected to DNA isolation for 16S-based microbiota analysis. Extended demographic and treatment data were recorded. Clinical outcomes were defined as: (i) implantation, confirmed by a positive hCG test, (ii) clinical pregnancy, and (iii) live birth (defined as the birth of a viable infant). Sequencing data were processed in mothur following established pipelines, and microbial composition (taxonomy) as well as microbial diversity (dissimilarity analyses) were determined using the open-source software R. A prediction model for implantation success was built using binary logistic regression based on abundance of putatively predictive microbial taxa.

**MAIN RESULTS AND THE ROLE OF CHANCE:**

This study suggests that vaginal microbial CSTs, alpha-diversity, and the ratio of dominant *Lactobacillus* species do not correlate in statistical terms or in a clinically meaningful manner with implantation and clinical pregnancy (as a surrogate for endometrial receptivity) or with live birth (as a surrogate for ongoing pregnancy viability). However, *Ureaplasma parvum* and *Lactobacillus iners* abundances were identified as negative predictors of embryo implantation, clinical pregnancy, and live birth. A subset of women colonized by these taxa experienced drastically reduced embryo implantation and completely failed to achieve clinical pregnancy or give birth to live offspring, suggesting a potential role of these organisms in implantation failure and reproductive outcome, independent of other influencing factors such as age, oestradiol levels, endometrial thickness etc.

**LARGE SCALE DATA:**

The raw sequencing data used for this manuscript are publicly available at the European Nucleotide Archive under accession number PRJEB107113.

**LIMITATIONS, REASONS FOR CAUTION:**

This study is a single-centre study warranting further validation cohorts. Given the variable nature of the vaginal microbiota, sample sizes need to be enlarged for better refinement of the analyses. Further, the underlying mechanistical basis of our findings is yet elusive and clinical translation has yet to be established.

**WIDER IMPLICATIONS OF THE FINDINGS:**

While this novel association warrants confirmation, the results caution against reliance on previously suggested CSTs as predictors, and highlight the need for refined, reproducible microbiome-based diagnostics in reproductive medicine.

**STUDY FUNDING/COMPETING INTEREST(S):**

Financial support was received from the University of Lübeck and the German Center of Infection Research. M.L., A.M., I.P., M.D., and J.R. declare no conflicts of interest. S.G. discloses personal fees from Organon outside the submitted work. T.K.E. discloses honoraria from Ferring; travel support from Ferring, Merck, Theramex, and Gedeon-Richter; and receipt of equipment/materials/laboratory analyses (to institution) from Arthrex, Besins, Merck, and Abbott outside the submitted work. N.H. discloses personal fees from Gedeon Richter, Ferring, and Merck. G.G. reports that his institution received grants or contracts from Besins, Merck, Abbott, Ferring, and Theramex. He has received personal consulting fees, support for travel fees and meeting attendance, and honoraria for lectures or educational events from Organon, Ferring, Merck, Gedeon-Richter, Theramex, Abbott, ReproNovo, Igyxos, OxoLife, Philipps, ReprodWissen, PregLem, Guerbet, Roche, IBSA, and Besins. He also received support for travel and meeting attendance from Merck, Organon, Ferring, Theramex, Gedeon-Richter, and Abbott. Additionally, he holds unpaid leadership positions as a member of the ESHRE Working Group on RIF, the ESHRE Working Group on clinical KPI, and the ESHRE guideline development group on ovarian stimulation. A.S.-M. reports consulting fees and speaker’s fees from Merck, Theramex, and Gedeon-Richter as well as travel support from Merck, Theramex, Gedeon-Richter, IBSA, Ferring, and MSD.

WHAT DOES THIS MEAN FOR PATIENTS? With all the existing medical knowledge and technology, the chance of getting pregnant from assisted reproduction is still rather low. Many patient-specific factors have been shown to influence the chance of pregnancy, and recently, the microbes living in the vagina have been identified to potentially influence assisted reproduction efficacy. Different bacteria have been suggested to prevent pregnancy while others should be more helpful. Tests are available to see whether those bacteria are present in the vagina.

Unfortunately, it is yet not precisely known which bacteria play a role in assisted reproduction efficacy, making it unclear whether microbiome tests should or should not be used to define the chance of pregnancy. We followed up on this question and could show that the previously suggested bacteria in fact do not provide the assumed information to predict pregnancy chances in assisted reproduction. As a conclusion, it may be assumed that the available microbial tests on bacterial community types to define pregnancy chance are not reliable.

Instead, the bacteria *Lactobacillus iners* and *Ureaplasma parvum* appear to predict failure in assisted reproduction. While vaginal bacteria should be further investigated to specify their role in efficacy of assisted reproduction, the exchange of bacteria which hinder assisted reproduction success with more helpful bacteria may be an available clinical method in the future.

## Introduction

Infertility is a global health issue affecting over 17% of the global population, summing up to 48 million couples affected worldwide, according to the World Health Organisation (WHO) (World Health Organization, 2023). Emotional sequels such as depression and anxiety (Szkodziak *et al.*, 2020) for affected couples are the major secondary burdens of infertility. ARTs, e.g. intra-uterine insemination and IVF, have assisted the birth of million babies since its introduction (Soini *et al.*, 2006). However, while ART is a viable and established treatment option for infertility, the success rate has remained rather low during the last decade, with ∼33% per treatment cycle (De Geyter *et al.*, 2018). The underlying reasons for treatment failure remain elusive in many cases (Gnoth *et al.*, 2011).

The microbial composition of the vagina of asymptomatic women has been suggested to contribute to infertility. Vaginal microbial communities are typically divided into community state types (CSTs) with communities of similar composition falling into same category. CSTs are largely described by the respective *Lactobacillus* species (e.g. *L. crispatus* or *L. iners*) dominating the microbiota or the absence of lactobacilli with enhanced diversity and other species (e.g. from the genera *Gardnerella* or *Atopobium*) appearing instead. Evidence suggests deviations from *Lactobacillus*-dominated bacterial communities being associated with infertility (Graspeuntner *et al.*, 2018; Chen *et al.*, 2025; Elahi *et al.*, 2025). In contrast to women conceiving spontaneously, women achieving pregnancy through IVF have a microbiota enriched in markers of vaginal dysbiosis (Lledo *et al.*, 2022). In this light, it appears likely that the microbiota may interfere with relevant outcomes of ART, from successful embryo implantation to delivery of a healthy newborn at term. Based on this assumption, there have meanwhile been several trials to identify microbial communities as predictive markers for ART success. A prominent study described the ratio of different *Lactobacillus* species as being putatively important for IVF outcome. It was suggested that *L. iners* was supportive for embryo implantation (Koedooder *et al.*, 2019). While these results achieved a high level of attention, the study has been called into question (Haahr *et al.*, 2019b; Gruteke *et al.*, 2021). Indeed, subsequent studies could not, or only partially, replicate the originally published association. Okwelogu *et al.* (2021) described *L. gasseri* being highly abundant in the vagina of women with pregnancy after IVF, while they, in contrast to Koedooder *et al.* (2019), found less *L. iners* in those women. This result has been supported by *L. iners* as such or the *L. iners*-dominated CST being associated with a lower positive pregnancy rate after IVF compared to *L. crispatus*-dominated CST (Wang *et al.*, 2021b; Guan *et al.*, 2023). This generally controversial picture has further been intricated by qPCR-based studies calling for *Prevotella bivia* and *Staphylococcus aureus* (Evans *et al.*, 2023) or more generalized abnormal vaginal microbial composition (Haahr *et al.*, 2019a) as negative impactors. A recent review, thus, emphasized the fact that the genital microbiota of females likely associates with IVF treatment outcome; however, there is still a lack of clear evidence which microbial factors are relevant in this context (Dube and Kar, 2022).

A number of factors may be relevant and explanatory for some of the divergent results between groups and the differences in diagnostic performance: different methodical setups are largely incomparable as they differ drastically in their resolution (Koedooder *et al.*, 2019; Okwelogu *et al.*, 2021; Wang *et al.*, 2021b; Guan *et al.*, 2023). Further, complexity of vaginal microbial community structure and its variability (Ravel *et al.*, 2011; Brooks *et al.*, 2017; France *et al.*, 2020) complicate studies on the vaginal microbiota (Robinson *et al.*, 2016). While vaginal microbiome diagnostics are already being marketed, the performance of these diagnostics in the context of a patient’s journey from fertility work-up to eventual treatments remains unknown.

We have, thus, performed a prospective, single-centre, well-controlled cohort study on the vaginal microbiome of 256 women undergoing frozen-thawed embryo transfer after IVF or intracytoplasmic sperm injection (ICSI) to unravel the key role of human vaginal bacteria in predicting treatment failure.

## Materials and methods

### Patients, ethics, sampling

The vaginal swab samples analysed herein stem from study subjects treated within a large, prospective, single-centre observational cohort study at the Department of Gynaecological Endocrinology and Reproductive Medicine of the University Hospital of Schleswig-Holstein, Campus Luebeck, Germany. The patients were recruited between May 2017 and March 2019. The study was prospectively registered at ClinicalTrials.gov (NCT03507673). Institutional review board approval was granted (Ethical Review Board of the University of Luebeck, reference number 18-005) and all patients provided written informed consent.

Infertile female patients (age 18–45 years) undergoing a frozen-thawed embryo transfer cycle in an anovulatory regimen (i.e. a cycle with transfer of an embryo following a previous oocyte retrieval, fertilization, and freezing of embryos) were recruited for the study. No preimplantation genetic testing for aneuploidy was performed. Inclusion criteria included no antimicrobial treatment for at least 6 weeks prior to embryo implantation and no symptoms indicative for genital infections. Each women contributed a single treatment cycle to the analysis performed herein. Endometrial build-up was achieved in all patients via administration of oral estradiol valerate (2 mg *per os*, tid; brand name Progynova^®^, Jenapharm GmbH & Co. KG, Jena, Germany) from Day 1 of menstruation onwards. On Days 13–15 of E2 administration, an appropriate tri-laminar endometrial build-up and the absence of a pre-ovulatory follicle were confirmed by an ultrasound scan. Moreover, suppression of ovulation was confirmed by serum progesterone determination in the anovulatory range. Patients were then scheduled for embryo transfer by initiating dydrogesterone (10 mg *per os* tid; brand name Duphaston^®^, Abbott Biologicals B.V., Weesp, Netherlands) as described in Neumann *et al.* (2022). Two weeks prior to embryo transfer, a swab (Copan UTM^®^: Universal Transport Medium for Viruses, Chlamydia, Mycoplasma, and Ureaplasma) was taken from the posterior fornix of the vagina under speculum visualization. The swab was taken not to be contaminated by contact with the vulva, the speculum, or the gloves of the physician. Swabs were frozen and stored at −80 °C directly after sampling. In all patients, exclusively dydrogesterone was administered by oral administration as the progestogenic drug to induce endometrial transformation and support early pregnancy.

### Clinical outcomes

A positive pregnancy test (i.e. serum hCG levels above the local centre’s reference range) 10–14 days after frozen embryonic transfer (FET) indicated implantation. Clinical pregnancy was defined by the presence of a foetal sac with a heartbeat on transvaginal sonography at GW 7 or later. Live birth was defined as the delivery of a living infant at or beyond the point of viability.

### DNA-isolation and amplicon generation

Vaginal swabs were processed using the DNeasy^®^ PowerSoil^®^ Pro Kit (Qiagen, Hilden, Germany) for DNA isolation. Therefore, samples were thawed and vortexed in the preservation medium and 250 µl of the medium was subjected to the kit following the recommended protocol including homogenization on a PowerLyzer (Qiagen). Isolated DNA was stored at −25°C in elution buffer until further processing.

To obtain amplicons from isolated DNA, PCR targeting V3/V4 hypervariable regions of 16S rRNA gene was performed. Each of the primers (Metabion, Planegg, Germany) contained a unique index sequence for further identification after sequencing as well as heterogeneity spacer regions for increased nucleotide diversity. For each of the samples the following master mix was prepared: 8.25 µl ultra-pure water, 5 µl high fidelity buffer, 0.5 µl dNTP mix (10 mM), 0.25 µl Phusion Hot Start II polymerase (2 U/µl), 5 µl of each primer (2 µM), and 1 µl sample DNA. Amplification was conducted under the conditions listed below: initial denaturation for 5 min at 94 °C followed by 30 cycles of denaturation for 30 s at 94 °C, annealing at 55 °C for 30 s and elongation at 72 °C for 1 min completed with 7 min of final elongation at 72 °C. In case of low concentrations of DNA in the sample, 5 µl of DNA was added. Obtained amplicons were stored at −25°C until further processing.

Next, 2% agarose (VWR International, Radnor, PA, USA) gel electrophoresis in 1× TAE buffer was carried out including RedSafe (iNTRON Biotechnology, South Korea) visualized via a transilluminator (Vilber Lourmat GmbH, Eberhardzell, Germany). The obtained images were analysed using Bio1D software (Vilber Lourmat), which identified DNA concentration via band intensity measurement with GeneRuler 100 bp DNA Ladder (Thermo Fisher Scientific, Waltham, MA, USA) as a referent marker. Based on measured intensities, amplicons were pooled at equimolar concentrations in a library which was subjected to further gel electrophoresis. Under UV light visualization, bands were cut out and DNA was extracted and purified via a MinElute^®^ Gel Extraction Kit (Qiagen). Subsequently, the amplicon library’s concentration was measured via quantitative PCR (qPCR) using NEBNext^®^ Library Quantification Kit for Illumina^®^ (New England Biolabs, Ipswich, MA, USA).

### Sequencing and data processing

Next-generation sequencing was performed based on the MiSeq^®^ platform (Illumina, San Diego, CA, USA). Therefore, the library was diluted to 4 nM and sequencing was performed according to instructions using end concentration of 10 pM including PhiX library v3 (Illumina) serving as a positive control.

Initial FASTQ generation was performed using BaseSpace Sequencing Hub platform (Illumina), thereafter obtained files were processed via mothur (version 1.43.0) (Schloss *et al.*, 2009). First, forward and reverse sequences were assembled in contigs if not exceeding 500 bp length or 12 bases homopolymers; next, Needleman–Wunsch pairwise alignment was performed against EzBioCloud database (Yoon *et al.*, 2017) followed by chimeric sequences removal using Vsearch algorithm (Rognes *et al.*, 2016). Taxonomic assignment of sequences was performed using EzBioCloud reference database with subsequent removal of eukaryotic, mitochondrial, and archaeal sequences. Operational taxonomic unit allocation was performed using taxonomic classification or 97% similarity rate cutoff.

### Statistics

A power analysis to assess required number of patients was conducted in G*Power (Faul *et al.*, 2009) version 3.1.9.7 as described within the Supplementary Materials and Methods. Analytical statistical calculations as well as graphical representation were performed on the basis of R (version 4.0.1) and R Studio (version 2022.12.0 + 353). To describe vaginal microbial community structure, CST allocation was done based on Euclidean hierarchical clustering. Visualization was displayed using heatmap created by the BoutrosLab.plotting package (P’ng *et al.*, 2019) and pie charts were created by the graphics package (Murrell, 2016). Furthermore, in order to characterize impact of various factors on vaginal microbial community relative abundances of identified species were compared using pairwise Wilcoxon rank-sum test by stats package (R Development Core, 2020) with corrections for false detection rate and visualized using graphics package (Murrell, 2016). Binomial data were compared using Fisher’s exact test or Chi-squared test. Alpha diversity was computed via Shannon’s diversity index and differences analysed using pairwise Wilcoxon rank-sum testing, vegan package (Oksanen *et al.*, 2019). Beta diversity was analysed through principle coordinates analysis on the basis of computing Bray–Curtis dissimilarities via the labdsv package (Roberts, 2013) with differences defined via permutational multivariate analysis of variance using distance matrices in the vegan package (Oksanen *et al.*, 2019). Identification of indicator species was performed by Linear Discriminant Analysis Effect Size provided by the Galaxy Project Platform (Segata *et al.*, 2011; Afgan *et al.*, 2018).

### Binary logistic regression to predict implantation outcome

In order to demonstrate the potential of novel predictors on the outcome of FET in the cohort, microbial predictor variables defined by LEFSE-Analysis and plasma dydrogesterone (DYD) values on day of implantation were included; the latter of these was recently shown to affect FET success (Neumann *et al.*, 2022). For some of the participants within this study, DYD-levels were not applicable, therefore missing values were imputed using Predictive Mean Matching (pmm), CART, and Lasso-imputation based on existing complete data for embryo implantation, age, and smoking. To choose the imputation mimicking best the data distribution of samples with existing DYD-values, the data distribution was checked and compared by histograms of the imputed versus the non-imputed data set.

Subsequently, binary logistic regression of the predictor variables was performed using implantation outcome (measured by hCG test) as the dependent variable. Comparison of the performance of the model against the baseline model identified terms significantly contributing to the model’s prediction. A probability threshold of 0.25 was set to separate participants with low likelihood of embryo implantation from those with high likelihood. Global estimates such as prediction accuracy and receiver operating characteristics curves were calculated. Further analysis of the low-likelihood group of the study cohort was enabled for the outcome parameters embryo implantation and live birth, and was followed by analyses on the vaginal microbial composition of those women based relative abundances and indicator species analysis (Cáceres and Legendre, 2009).

## Results

In this study, 266 women undergoing FET were included, and data for microbiome analysis were available in 256 cases. Thus, all analyses and results presented herein are based on this number. Of the 256 women, 116 (45.3%) had successful embryo implantation as shown by a positive hCG-test while 140 women (54.7%) remained negative for pregnancy. The first analysis compared demographic data and clinical variables of the cohort. The given variables and criteria matched well between women with and without embryo implantation following IVF (Table 1). The same data stratified for clinical pregnancy and live birth within the cohort are provided in Supplementary Tables S1 and S2.

**Table 1.** Demographics and treatment variables of the study cohort.

	Positive hCG (Implantation; n = 116)	Negative hCG (no Implantation; n = 140)	*P*-values (test)
**Age at day of embryo transfer (years)**	33.1 ± 4.08 [32.3–33.8]	33.6 ± 4.04 [32.9–34.3]	n.s. (*t*)
**Patients height (cm)**	167 ± 4.08 [166–168]	168 ± 4.04 [167–169]	n.s. (*t*)
**Patients weight (kg)**	67.1 ± 11.7 [65.5–69.8]	69.6 ± 15.3 [67.1–72.2]	n.s. (*U*)
**Duration of infertility (months)**	39.9 ± 14.4 [36.7–43.2]	43.6 ± 16.8 [40.4–46.9]	n.s. (*U*)
**Caucasian **	96/116	119/140	n.s. (χ²)
**Main cause of infertility**			n.s. (Fisher)
Male subfertility	71/116	74/140	
Idiopathic	17/116	24/140	
Tubal factor infertility	19/116	25/140	
Polycystic ovarian syndrome	5/116	8/140	
other	1/116	7/140	
**Number of patients with pre-existing diseases**			n.s. (Fisher)
No pre-existing diseases	81/116	96/140	
(Latent) Hypothyroidism	24/116	24/140	
Diabetes mellitus type 2	0/116	4/140	
Others (e.g. Asthma bronchiale; Hypertonus; Migraine)	11/116	16/140	
**Number of patients with previous surgery**			n.s. (Fisher)
No prior surgery	73/116	79/140	
Laparoscopic surgery	18/116	23/140	
Hysteroscopic surgery	1/116	6/140	
Hysteroscopy and laparoscopy	10/116	10/140	
Cesarean section	3/116	11/140	
Curettage	7/116	4/140	
Conization	4/116	7/140	
**Number of patients with regular menstrual cycle**	93/116	109/140	n.s. (χ²)
**Number of patients smoking**	19/116	31/140	n.s. (χ²)
**Number of patients with polycystic ovarian syndrome**	15/116	19/140	n.s. (χ²)
**Endometriosis, any stage**	5/116	10/140	n.s. (Fisher)
**Pelvic inflammatory disease**	3/116	8/140	n.s. (Fisher)
**Number of patients with previous ectopic pregnancy**	5/116	14/140	n.s. (Fisher)
**Nulligravida**	46/116	71/140	n.s. (χ²)
**Number of patients with previous miscarriage**	30/116	29/140	n.s. (χ²)
**Number of embryo transfers before inclusion into study**	1.30 ± 1.39 [1.05–1.56]	1.73 ± 2.37 [1.33–2.13]	n.s. (*U*)
**Number of 2PNs cryopreserved at prior ovum pick-up**	8.35 ± 5.28 [7.32–9.39]	7.84 ± 5.24 [6.92–8.76]	n.s. (*U*)
**Number of embryos transferred at FET**	1.28 ± 0.453 [1.20–1.37]	1.32 ± 0.469 [1.24–1.40]	n.s. (*U*)
**Proportion of patients with single embryo transfer**	83/116	95/140	n.s. (χ²)
**Endometrial thickness (mm) at last monitoring**	9.29 ± 5.14 [8.30–10.3]	8.78 ± 1.86 [8.46–9.10]	n.s. (*t*)
**Add ons**			
Embryo glue	9/116	20/140	n.s. (χ²)
Assisted hatching	2/116	3/140	n.s. (χ²)
**Plasma dydrogesterone at FET (ng/ml) (n = 186)**	1.46 ± 1.08 [1.23–1.70]	1.34 ± 1.52 [1.17–1.52]	n.s. (*U*)
**Plasma dihydrodydrogesterone at FET (ng/ml) (n = 186)**	39.6 ± 25.4 [34.1–45.1]	35.7 ± 20.2 [31.7–39.7]	n.s. (*U*)
**Serum progesterone at FET (ng/ml) (n = 88)**	0.116 ± 0.101 [0.0841–0.149]	0.116 ± 0.102 [0.0860–0.145]	n.s. (*U*)
**Serum estradiol at FET (pg/ml) (n = 217)**	227 ± 102 [206–247]	202 ± 86.7 [186–218]	n.s. (*U*)
**Plasma dydrogesterone at hCG (ng/ml) (n = 154)**	1.70 ± 1.10 [1.45–1.96]	1.62 ± 1.13 [1.37–1.87]	n.s. (*U*)
**Plasma dihydrodydrogesterone at hCG (ng/ml) (n = 155)**	46.9 ± 25.7 [40.9–52.9]	45.6 ± 28.0 [39.4–51.7]	n.s. (*U*)
**Serum progesterone at hCG (ng/ml) (n = 96)**	2.21 ± 6.02 [0.684–3.74]	0.430 ± 1.78 [−0.192 to 1.05]	n.s. (*U*)
**Serum estradiol at hCG (pg/ml) (n = 189)**	403 ± 241 [352–453]	280 ± 178 [245–316]	*** (*U*)

Depicted are mean ± SD or numbers (n/N), as appropriate. Statistics: *t*, Student’s *t*-test; *U*, Mann–Whitney *U*-test; χ^2^, chi-squared test; Fisher, Fisher’s exact test.

*** =*P* < 0.001. FET, frozen embryo transfer.

### Generalized microbial patterns do not reliably predict IVF outcomes

**Figure 1.**
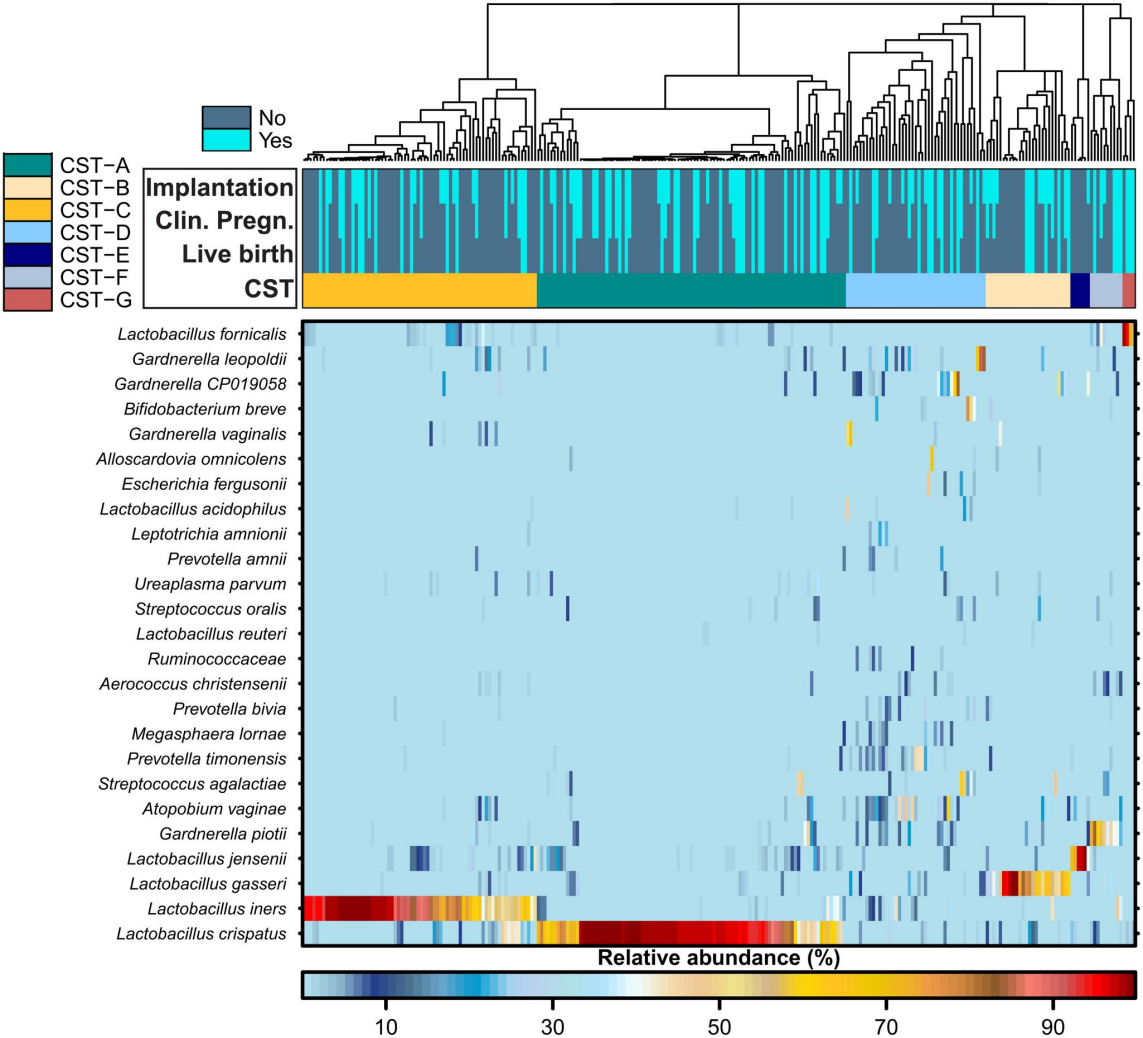
**Heatmap with hierarchical clustering depicts vaginal bacterial composition and community state type assignment.** Seven community state types (CSTs) are described based on the relative abundance of the 25 most present bacterial taxa classified to species level. The CSTs are represented by dominance of the *Lactobacillus* species *L. crispatus* (CST-A), *L. gasseri* (CST-B), *L. iners* (CST-C), *L. jensenii* (CST-E), and *L. fornicalis* (CST-G), the dominance of *Gardnerella piotii* (CST-F) or a rather diverse type of communities (CST-D). Implantation, clinical pregnancy, live birth, and CSTs are given as covariates for each participant below the cladogram of the hierarchical clustering. Clin. Pregn, clincical pregnancy; CST, community state type.

As a conventional method to understand the community structure of the vaginal microbiota of the present cohort, a heatmap with hierarchical clustering of samples was constructed, both based on the relative abundance of the 25 most abundant bacterial taxa when classified to species level (Fig. 1). Based on the clustering, seven separate CSTs were identified as being either dominated by one definable species of the genera *Lactobacillus* or *Gardnerella* (CST-A, CST-B, CST-C as well as CST-E, CST-F, CST-G) or presenting a variable community composition (CST-D). It may be pointed out that a CST dominated by *L. fornicalis* was identified in a small fraction of women (1.6%) and that depiction of enhanced resolution within the *Gardnerella* genus impacts CST definitions. Other CSTs match the previously established CST definitions. Importantly, implantation, clinical pregnancy, and live birth were equally distributed across the identified CSTs (Fig. 1).

**Figure 2.**
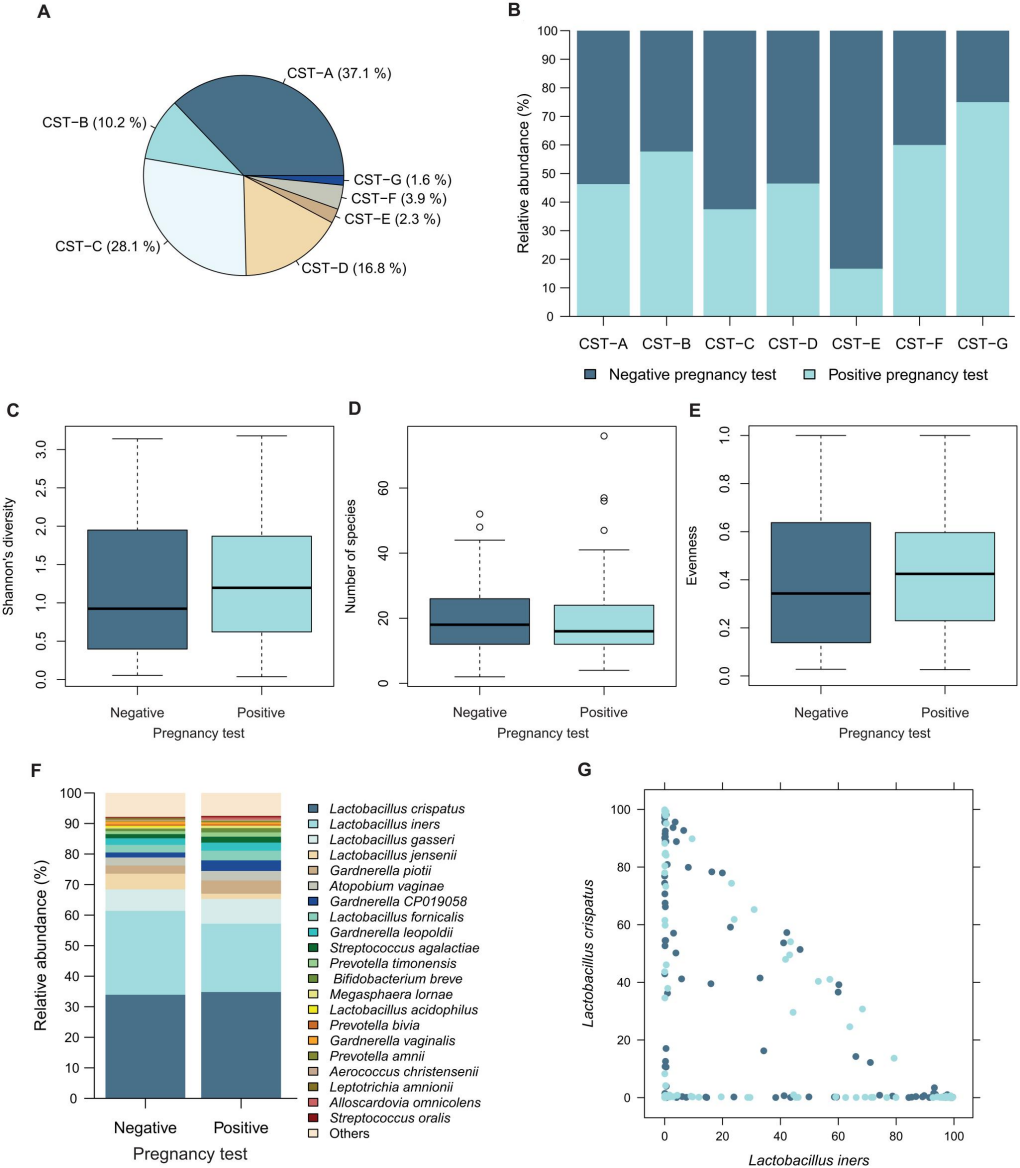
**Global measures of microbial communities fail to contribute to the explanation of embryo implantation in univariate analyses.** The majority of women participating in this study displayed a vaginal bacterial community defined as CST-A, CST-B, CST-C, and CST-D, while other CSTs account for minor numbers of community description at the sampling time point (**A**). Variations in IVF-success between CSTs were not statistically significant (**B**) and neither were alpha diversity measures (**C–E**). Relative abundance of major taxa of the data set (**F**) as well as depiction of *L. crispatus* vs *L iners* relative abundances (**G**) failed to explain the positive versus negative outcome of IVF in this study cohort. CST, community state type. Statistics: Fisher’s exact test (panel B) and Wilcoxon rank-sum test (panels C–E).

The majority of women participating in this study were colonized by one of the two *Lactobacillus*-dominated CST-A (37.1%) or CST-C (28.1%). While CST-D (16.8%) and CST-B (10.2%) displayed a lower presence in our study cohort, the remaining CSTs (CST-F, CST-E, CST-G) made up only for 3.9%, 2.3%, and 1.6%, respectively (Fig. 2A). Observed variations in implantation rate between CSTs (e.g. enhanced in CST-G) were not statistically significant (Fig. 2B). It may be noted that when addressing alpha-diversity measures (Shannon’s diversity (Fig. 2C), the number of observed species (Fig. 2D), the evenness (Fig. 2E)), and the major observed taxa on the species level (Fig. 2F), showed no obvious differences between women with or without positive IVF outcomes. As the ratio of relative abundances of *L. iners* versus *L. crispatus* has been suggested to influence IVF treatment, Fig. 2G depicts respective values but we failed to find this ratio to be associated with implantation in the present cohort. Similar results considering clinical pregnancy and live birth as stratifying variables are shown in Supplementary Figs S1 and S2.

### *Ureaplasma parvum*, *Lactobacillus iners*, and *L. fornicalis* are associated with IVF outcome and their abundance values guide precise prediction of negative IVF outcome

**Figure 3.**
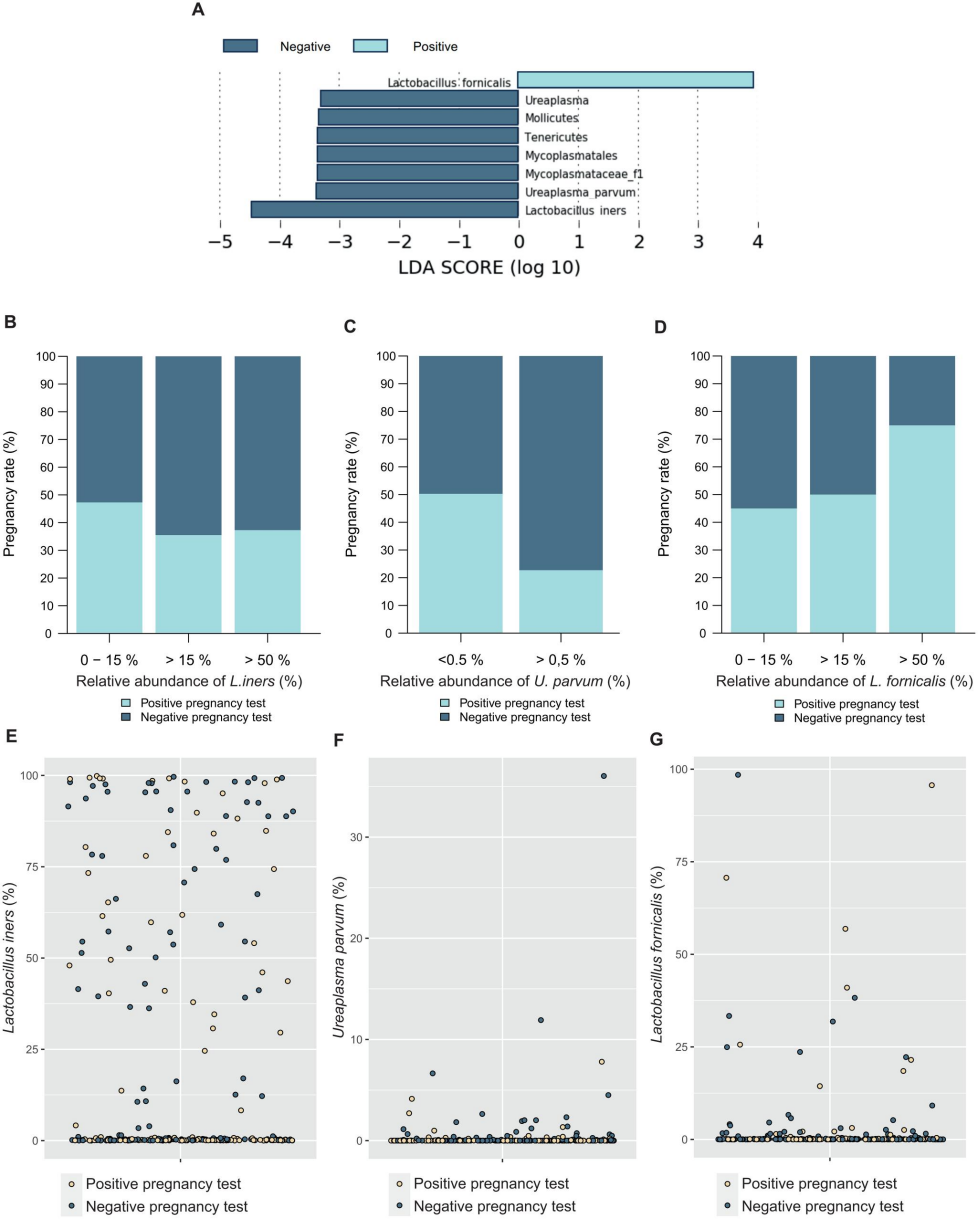
**Linear discriminant analysis effect-size-assisted identification of bacterial predictors enables analysis of their association to embryo implantation.** Setting effect size threshold to 3, we identified the species *L. fornicalis*, *U. parvum*, and *L. iners* to be significantly associated with outcome in IVF treatment (**A**). Defining groups based on relative abundances of those three species individually showed that higher values for *L. iners* yield moderately lower levels of pregnancy rate (**B**), while relative abundance of > 0.5% for *U. parvum* associates with drastically decreased treatment success (**C**). On the contrary, increasing relative abundance of *L. fornicalis* associated with increased embryo implantation (**D**). Panels (**E–G**) depict abundance versus result of pregnancy test for each woman. LDA, linear discriminant analysis; *L. iners*, *Lactobacillus iners*; *U. parvum*, *Ureaplasma parvum*; *L. fornicalis*, *Lactobacillus fornicalis*.

LEfSe-Analysis was applied to identify bacterial taxa being associated with the outcome of IVF treatment. When setting the minimum threshold for the LDA score to 3, *L. fornicalis* was positively associated with embryo implantation, while in contrast, *L. iners* and *U. parvum* were associated with absence of pregnancy following IVF (Fig. 3A). Indeed, a minimum of just 0.5% relative abundance of *U. parvum* was sufficient to decrease embryo implantation from 50.2% without *U. parvum* to 22.7% in women undergoing FET (Fig. 3B). In contrast, subsequent analyses identified nearly 75% of women as having a positive hCG test when relative abundance of *L. fornicalis* is above 50% (Fig. 3D). Complete respective analyses on *L. iners*, *U. parvum*, and *L. fornicalis* are depicted in Fig. 3B–G, and Supplementary Figs S3 and S4, yielding similar results also for clinical pregnancies and live birth (e.g. 24.8% vs 9.1% live birth rate, respectively) (Supplementary Fig. S4B).

**Figure 4.**
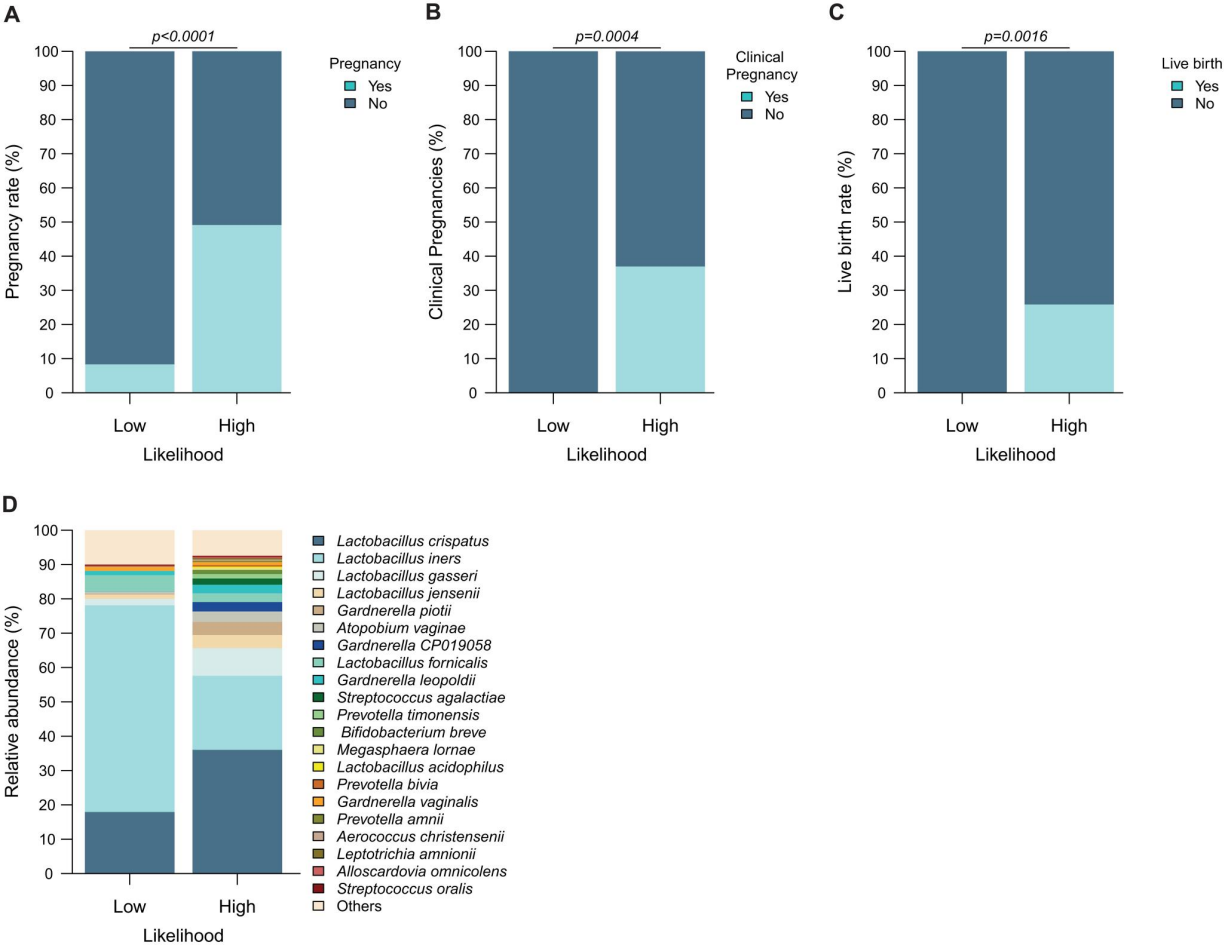
**Logistic regression-enabled prediction model based on relative abundance of *L. iners*, *U. parvum*, and *L. fornicalis* identifies a group of women with a very low chance of achieving implantation success.** The prediction identifies women of the low-likelihood group to have an implantation chance of only 5% compared to the rest of the women with high pregnancy chance, i.e. having 50% success rate (**A**). None of the women from the low-likelihood group achieved clinical pregnancy (**B**) or live birth (**C**). Women predicted to have low likelihood for embryo implantation display a microbial community over-representing *L. iners* as the dominant bacterial species compared to other women (**D**). Statistics (panels A–C): Fisher’s exact test.

Further, a binary logistic regression analysis was constructed using the relative abundance values of the three potential predictor species to evaluate their potential in a prediction model. As dydrogesterone levels are a predictor for ongoing embryo implantation in an HRT-FET progestin monotherapy protocol, this variable was incorporated into the prediction model (after performing predictive mean matching imputation for missing values of the dydrogesterone variable; Supplementary Fig. S5). It appears that the interaction between *U. parvum* and *L. iners* is significantly associated with treatment outcome in this approach (*P*-value: 0.0160). The same significant association with treatment outcome is the case for the interaction between *U. parvum* and dydrogesterone (*P*-value: 0.0400), as well as *U. parvum* with *L. fornicalis* (*P*-value: 0.0320). From the fitted model, computation of the log-likelihood aimed to predict outcome of the FET cycle by the three potential predictor species. Using 0.5 as threshold for the prediction model yielded only a moderate prediction accuracy of 60.2% overall. In contrast to the low overall prediction quality of the model (see receiver operating curves for positive and negative prediction in Supplementary Fig. S6), a precise negative prediction for women below the prediction threshold of 0.25 when predicting outcome of FET was identified. While in this specific group of 24 women, 2 still had embryo implantation (Supplementary Table S3, Fig. 4A), none of them achieved clinical pregnancy or live birth (Supplementary Table S3, Fig. 4B and C). This group of women, thus, is named the ‘low-likelihood group’ and the group of women with a probability-value of >0.25 was named the ‘high-likelihood group’.

**Figure 5.**
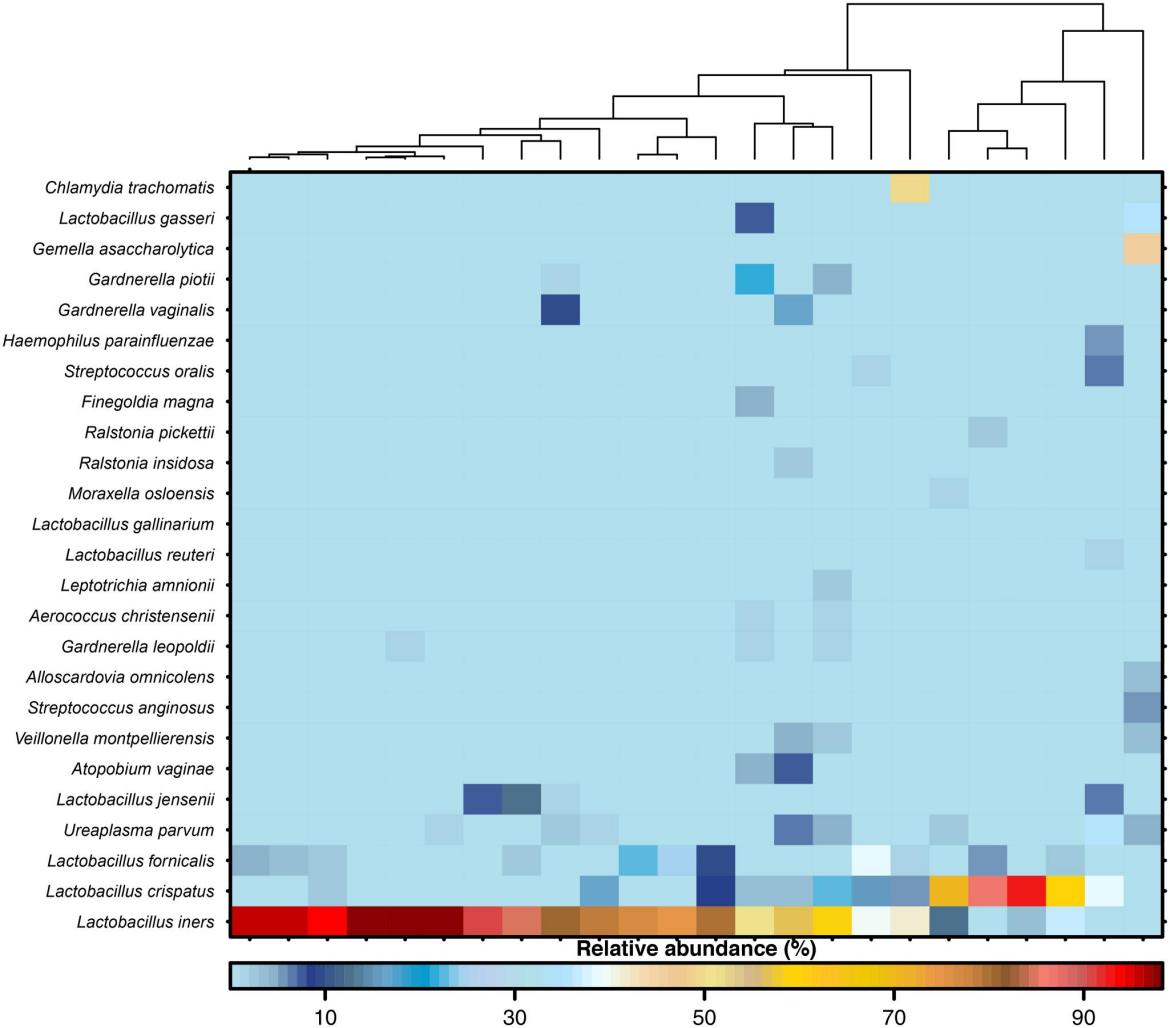
**Sample-by-sample depiction of women with a predicted low likelihood of achieving embryo implantation.** Bacterial communities of majority of women of the low-likelihood group is dominated by *L. iners* while other lactobacilli are sparse. The low-likelihood group includes a case of apparent *C. trachomatis* infection.

Indicator species analysis reinforced *U. parvum* as the major taxon being apparent in the low-likelihood group (*P*-value: 0.005). Furthermore, 21 of those 24 women harboured *U. parvum* and those 21 women carried 94.08% of all present respective reads within the complete data set, while the 230 women who were part of the low-likelihood group shared the remaining 5.92% of *U. parvum* reads (Table 2). It is noteworthy, that only indicators for the low-likelihood group were identified with this analysis, while no indicators for the women of the high-likelihood group were apparent. In addition, abundance analysis showed increased *L. iners* (Fig. 4D) for the low-likelihood group, with the majority of these samples belonging to the *L. iners*-dominated CST-C (Fig. 5). Among further species, a case of *C. trachomatis* infection within the low-likelihood group (Fig. 5) was apparent. Of further interest, when computing the low-likelihood group based on the microbial predictors only (Supplementary Table S4), *C. trachomatis* was identified as an indicator (Supplementary Table S5) and was restricted almost completely to the low-likelihood group (*P*-value: 0.01) in this analysis.

**Table 2.** Most prevalent taxa identified as indicators for the low-likelihood group predicted with plasma dydrogesterone: values of women undergoing IVF-treatment.

Taxon	A	B	*P*-value
*Ureaplasma parvum*	0.941	0.8750	**
*Lactobacillus coleohominis*	0.662	0.250	*
*Veillonella montpellierensis*	0.907	0.208	*

Indicator species analysis shows the taxa which are significantly associated with low-likelihood of embryo implantation from IVF treatment. A, ratio of total number reads of the respective taxon appearing in low-likelihood group. B, ratio of samples within the low-likelihood group displaying reads of the respective taxon. Statistics: Permutation test within r-package ‘indicspecies’.

* =*P* < 0.05;

** =*P* < 0.01.

## Discussion

IVF treatment suffers from low success rates of only around one in every three attempts being accomplished by implantation of an embryo into the uterus (De Geyter *et al.*, 2018). While most failures may be attributed to embryonic origin, such as embryonic chromosomal aneuploidy (Scott *et al.*, 2012; Tiegs *et al.*, 2021; Wang *et al.*, 2021a; Yang *et al.*, 2021), a significant proportion of repetitive treatment failures, even in young couples with a good prognosis at treatment outset, remain enigmatic. The notion that the microbiota of the female urogenital tract could serve as an indicator of treatment success has garnered significant attention, leading to increased research on vaginal microbiota in the context of reproduction (e.g. as recently reviewed by Gao *et al.* (2022)). While there is substantial evidence suggesting a link between fertility and the vaginal microbiome (e.g. Graspeuntner *et al.*, 2018; Koedooder *et al.*, 2019; Hao *et al.*, 2021; Lüth *et al.*, 2022), no consensus has been reached regarding causal relationships (Okwelogu *et al.*, 2021; Wang *et al.*, 2021b; Guan *et al.*, 2023). This lack of consensus raises questions about the validity of current testing strategies, positioning diagnostic microbiome applications in this field more as a future prospect rather than a ready-to-use clinical solution (García-Velasco *et al.*, 2020). This study, thus, completed a trial on identifying vaginal microbial communities in women undergoing frozen-thawed embryo transfer and relating this data to treatment success. Records of a large variety of clinical variables with the potential to impact the microbiological data and embryo implantation were assessed. For all those parameters, the cohort appears to be well matched between women with and without embryo implantation.

While an association of the ratio of *L. iners*/*L. crispatus* has been originally described with a high abundance of *L. iners* being supportive to ART implantation success (Koedooder *et al.*, 2019), this has not been recapitulated ever since by other independent studies. Confirming the caution with this original finding, this study was also not able to confirm that women with high *L. iners* but low *L. crispatus* abundance would have a higher likelihood for getting pregnant. Further findings from studies addressing conditions associated with vaginal tract health (such as transmission of sexually transmitted infections or indications for preterm birth) suggest higher diversity to be a detrimental microbial factor linked to infertility (Graspeuntner *et al.*, 2018; Chen *et al.*, 2025; Elahi *et al.*, 2025) and implantation success in assisted reproduction (Fu *et al.*, 2020). However, this seems to not apply in the light of this study and other recently published data (Wang *et al.*, 2021b). Variations between CSTs in implantation success appear to be rather minor for abundant CSTs in the present study while they are not of statistically significant relevance for lower abundance CSTs. This again may be a very important factor considering unequal distribution of CSTs among populations with varying ethnic compositions (Ravel *et al.*, 2011; Borgdorff *et al.*, 2017; Roachford *et al.*, 2022), as it rules out a link between treatment success and easy-to-handle community assignment data, as our results highlight.

In contrast to assessment of global microbial measures, this study provides evidence for three species being associated with treatment outcome: *L. iners* and *U. parvum*, as being negatively associated, and the newly CST-forming *L. fornicalis*, as being positively associated. This is of particular relevance due to several considerations. First, *L. iners* has been addressed as a negative predictor for implantation success already by other colleagues (Karaer *et al.*, 2021; Okwelogu *et al.*, 2021; Wang *et al.*, 2021b; Guan *et al.*, 2023). Second, while *L. iners* belongs to the most common bacterial species in the human vagina, also *U. parvum* is found in approximately up to one-third of the European population (Leli *et al.*, 2018; Rak *et al.*, 2022), though in typically low relative abundance (Hong *et al.*, 2021). *Ureaplasma* prevalences are even higher in the USA (Ramaloko *et al.*, 2025). Thus, those two species may have high usability for building prediction models for the treatment outcome, and their high prevalence may (in part) serve as explanation for the low success of ART within a segment of the total population. Third, the positive association of *L. fornicalis* highlights the need to expand knowledge on the presence of yet undescribed and understudied species and what their impact on the function of the vaginal microbiome may be.

The three indicator species served as covariates to produce a binary logistic regression model on the dependent variable implantation. As with other models published previously (Wang *et al.*, 2021b), this model does not have the capacity of explaining treatment success well for the study population globally. But, it has a very promising feature of defining a low-likelihood-group of women for which a negative prediction is highly accurate. The logistic regression clearly supports the indicator species analysis identifying *U. parvum* as the main driver of the definition of the low-likelihood group. This appears to be of great importance because the role of *U. parvum* in the human vagina is under heavy debate: whether it is detrimental to vaginal health or part of the commensals in the human vaginal microbiome (Hong *et al.*, 2021). It should be pointed out that already the pure presence of *U. parvum* is associated with a decrease of the implantation success to nearly 20%, while the entire study cohort displays an implantation rate of ca. 50%. However, it appears to be increasingly detrimental if *U. parvum* coincides with the presence and high abundance of *L. iners*, which is highly overrepresented in the low-likelihood group compared to the remaining women.

Given the herein presented data, it appears likely that specific microbes need to interact or at least be present together to display a negative effect on implantation success in an ART. The fact that *U. parvum* had been a non-observed factor in this process may partially explain why studies so far were contradictory to each other. However, it needs to considered as well that several low-prevalence or low-abundance microbes are described to hinder ART success (Kong *et al.*, 2020; Xu *et al.*, 2020; Karaer *et al.*, 2021), which may also mean that a wider combination of identified bacteria from the vagina may contribute to a better (negative) prediction. The fact that *U. parvum* is also considered a pathogen increasing baseline inflammatory markers in colonized women (De Seta *et al.*, 2019) may explain its capacity to hinder embryo implantation. However, available data currently do not demonstrate that antibiotic treatment eradication will solve the problem. Detrimental effects of antibiotic treatment on the microbiota and experiences from other *U. parvum*-associated diseases resulted in cautious to negative recommendations for direct antibiotic therapies (Horner *et al.*, 2018). However, the current findings open the door for future clinical interventional studies focussing on modulation of the vaginal microbiota before assisted reproduction trials.

The prediction model’s performance was enhanced when introducing dydrogesterone levels as a predictor variable. This sheds light on a very important point for the likelihood of successful ART: there are various factors known which influence assisted reproduction outcome. While this study puts emphasis on the vaginal microbiome, the microbiome as such can be crucial but only be a partial variable in explaining embryo implantation, clinical pregnancy, and live birth rates. Larger studies are needed to delineate in more detail all known influencing variables and their interdependence with the vaginal microbiome to better understand what portion of ART success is influenced by the microbiome.

### Limitations and reasons for caution

This study is a single-centre study. It remains to be proven whether the herein presented data are reproducible at other centres. This ultimately means that the data are warranting further validation in multicentre cohorts. Sample sizes are an issue in microbiota analyses from vaginal samples. Given the variable nature of the vaginal microbiota, increasing sample sizes will lead to better refinement of the analyses. In addition, technical challenges in 16S rRNA gene-based testing for *U. parvum* apply to microbiota analyses. In particular, false negative data due to low abundance combined with limited sequencing depth may theoretically skew the results. Given the apparent importance of *U. parvum*, it may prove valuable to add *U. parvum* testing using certified diagnostic testing to comparable study settings. Considering the study design, data on participant’s sexual behaviour, vaginal douching, and probiotics usage are missing. While this should not introduce bias to the study based on the expected adherence of patients undergoing IVF treatment to generalized practical guidelines, it should be assessed in upcoming studies to enable refinement of the provided data analyses. Last, an underlying mechanistical basis of our findings was not a focus of this work and remains elusive but important for translation to practical guidelines and treatment strategies during IVF treatment.

When analysing the microbial communities of the global study population, this study identifies a picture which reflects the central European study population with about 80% of women presenting with *Lactobacillus*-dominated communities while only a minority display rather diverse (CST-D) or *Gardnerella*-dominated (CST-F) communities. Whenever discussing the impact of the microbial communities on vaginal and reproductive health, one should keep in mind that such CST-distributions may vary between studies, especially when different ethnicities are among the participating women. Of particular note is that this study describes a CST which is dominated by *L. fornicalis*, a described valid species (Dicks *et al.*, 2000) that is currently under debate, as the type strain has been lost (Ribeiro *et al.*, 2020). This is also the reason for not considering the standardized VALENCIA-method (France *et al.*, 2020) for the description of CSTs as *L. fornicalis* is not among the species selected as central in the CST assignment. We, however, suggest that our CST definition largely aligns to those of VALENCIA, except for CST-G.

## Conclusions

This prospective study does not support the clinical relevance of previously proposed vaginal microbial CSTs, alpha-diversity measures, or ratios of dominant *Lactobacillus* species as predictors of ART outcome. Instead, a distinct subgroup of women colonized by *Lactobacillus iners* in combination with *Ureaplasma parvum* exhibited markedly reduced implantation rates and, in some cases, complete failure to achieve clinical pregnancy or live birth, independent of established confounders, including maternal age. These results suggest that broad, community-based microbiome classifications may be insufficient for clinical risk stratification, whereas taxon-specific approaches may better capture biologically relevant associations with reproductive outcome. While external validation and mechanistic studies are required before clinical implementation, our data caution against the uncritical use of current CST-based microbiome diagnostics in ART, some of which are commercially available and routinely used. The results also underscore the need for robust, reproducible, and biologically informed biomarkers to guide future translational research.

## Supplementary Material

hoag018_Supplementary_Data

## Data Availability

The raw sequencing data used for this manuscript are publicly available at the European Nucleotide Archive under accession number PRJEB107113 including relevant metadata. The mothur script is provided within the Supplementary Materials and Methods. Only freely available R codes were used to generate the data analyses for this manuscript.
