# Supplementary material for: Vaginal microbial community state types fail to predict IVF outcomes, whereas Ureaplasma parvum and Lactobacillus iners are negative predictors of implantation, clinical pregnancy, and live birth
Source: Hum Reprod Open. 2026 Mar 5;2026(2):hoag018. doi: 10.1093/hropen/hoag018 (PMC13037765; doi:10.1093/hropen/hoag018)
Supplement: hoag018_Supplementary_Data [file hoag018_supplementary_data.docx]

**Vaginal microbial community state types fail to predict IVF outcomes, whereas *Ureaplasma parvum* and *Lactobacillus iners* are negative predictors of implantation, clinical pregnancy, and live birth**

Simon Graspeuntner, Mariia Lupatsii, Noemi Hamala, Antonia Masuch, Marion Depenbusch, Iris Pfeffer, Askan Schultze-Mosgau, Tanja Eggersmann, Jan Rupp, Georg Griesinger

**Supplementary Data**

**Supplementary Table S1: Demographics and treatment variables of the study cohort stratified by clinical preganancy.**

|  | Clinical pregnancy  (n = 86) | No Clinical pregnancy  (n = 170) |
| --- | --- | --- |
| Age at day of embryo transfer (years) | 33.1 ± 3.69  [32.3 – 33.9] | 33.5 ± 4.24  [32.9 – 34.2] |
| Patients height (cm) | 167 ± 6.38  [165 – 168] | 168 ± 6.70  [167 – 169] |
| Patients weight (kg) | 67.2 ± 11.6  [64.7 – 69.7] | 69.5 ± 14.7  [67.3 – 71.8] |
| Duration of infertility (months) | 40.5 ± 14.7  [36.5 – 44.6] | 42.7 ± 16.3  [39.8 – 45.5] |
| Caucasian (%) | 71/86 (82.6%) | 144/170 (84.7%) |
| Causes of infertility (%) |  |  |
| - Male subfertility | 53/86 (84.9%) | 103/170 (60.6%) |
| - Idiopathic | 15/86 (17.4%) | 26/170 (15.3%) |
| - Tubal factor infertility | 15/86 (17.4%) | 33/170 (19.4%) |
| - Polycystic ovarian syndrome | 4/86 (4.7%) | 17/170 (10.0%) |
| - others | 4/86 (4.7%) | 11/170 (6.5%) |
| Number of patients with pre-existing diseases (%) |  |  |
| - No pre-existing diseases | 60/86 (69.8%) | 117/170 (68.8%) |
| - Hypothyroidism | 19/86 (22.1%) | 29/170 (17.1%) |
| - Diabetes mellitus type 2 | 0/61 (0%) | 4/170 (2.4%) |
| - Others (e.g. Asthma; Hypertonus) | 7/86 (8.1%) | 20/170 (11.8%) |
| Number of patients with previous surgery (%) |  |  |
| - No prior surgery | 53/86 (61.6%) | 99/170 (58.2%) |
| - Laparoscopic surgery | 11/86 (12.8%) | 30/170 (17.6%) |
| - Hysteroscopic surgery | 1/86 (1.2%) | 6/170 (3.5%) |
| - Hysteroscopy and laparoscopy | 7/86 (8.1%) | 13/170 (7.6%) |
| - Cesarean section | 3/86 | 11/170 (6.5%) |
| - Currettage | 7/86 (8.1%) | 4/170 (2.4%) |
| - Conisation | 4/86 (4.7%) | 7/170 (4.1%) |
| Number of patients with regular menstrual cycle (%) | 70/86 (81.4%) | 132/170 (77.6%) |
| Number of patients smoking (%) | 12/86 (14.0%) | 38/170 (22.4%) |
| Number of patients with polycystic ovarian syndrome (%) | 9/86 (10.5%) | 25/170 (14.7%) |
| Number of patients with endometriosis (%) | 3/86 (3.5%) | 12/170 (7.0%) |
| Number of patients with previous ectopic pregnancy (%) | 5/86 (5.8%) | 14/170 (8.2%) |
| Number of patients with previous miscarriage (%) | 23/86 (26.7%) | 36/170 (21.2%) |
| Number of patients with no previous embryo transfer before inclusion into analysis (%) | 24/86 (27.9%) | 56/170 (32.9%) |
| Number of 2PNs cryopreserved at prior ovum pick-up | 8.82 ± 5.33  [7.62 – 10.0] | 7.68 ± 5.19  [6.85 – 8.52] |
| Number of embryos transferred | 1.30 ± 0.462  [1.20 – 1.40] | 1.31 ± 0.463  [1.24 – 1.38] |
| Proportion of patients with top quality embryo transfer (%) | 67/86 (77.9%) | 94/170 (55.3%) |
| Endometrial thickness (mm) at embryo transfer | 9.21 ± 5.83  [7.90 – 10.5] | 8.91 ± 1.98  [8.60 – 9.21] |
| Pelvic inflammatory disease | 2/86 (2.3%) | 9/170 (5.3%) |
| Add ons |  |  |
| - Embryo glue | 8/86 (9.3%) | 21/170 (12.4%) |
| - Assisted hatching | 2/86 (2.3%) | 3/170 (0.6%) |
| Dydrogesterone at ET (ng/ml) (n =186) | 1.38 ± 0.910  [1.15 – 1.60] | 1.41 ± 1.02  [1.23 – 1.59] |
| Dihydrogesterone at ET (ng/ml) (n =186) | 37.5 ± 21.6  [32.1 – 42.9] | 37.5 ± 23.4  [33.3 – 41.7] |
| Progesterone at ET (ng/ml) (n = 88) | 0.131 ± 0.113  [0.0885 – 0.173] | 0.108 ± 0.0942  [0.0836 – 0.133] |
| Estradiol at ET (pg/ml) (n = 216) | 231 ± 100  [208 – 255] | 204 ± 90.1  [189 – 219] |
| Dydrogesterone at hCG (ng/ml) (n = 154) | 1.69 ± 1.07  [1.41 – 1.97] | 1.64 ± 1.15  [1.41 – 1.87] |
| Dihydrogesterone at hCG (ng/ml) (n = 155) | 47.5 ± 25.2  [40.9 – 54.2] | 45.4 ± 27.8  [39.8 – 51.0] |
| Progesterone at hCG (ng/ml) (n = 96) | 2.62 ± 6.50  [0.811 – 4.43] | 0.352 ± 1.57  [-0.125 – 0.829] |
| Estradiol at hCG (pg/ml) (n = 189) | 425 ± 256  [363 – 488] | 290 ± 177  [258 – 322] |

Depicted are mean and SD or numbers and proportions, as appropriate.

**Supplementary Table S2: Demographics and treatment variables of the study cohort stratified by live birth.**

|  | Live birth  (n = 60) | No live birth  (n = 196) |
| --- | --- | --- |
| Age at day of embryo transfer (years) | 33.1 ± 3.45  [32.3 – 34.0] | 33.4 ± 4.24  [32.8 – 34.0] |
| Patients height (cm) | 166 ± 6.58  [164 – 168] | 168 ± 6.57  [167 – 169] |
| Patients weight (kg) | 67.7 ± 12.3  [64.5 – 70.9] | 69.1 ± 14.2  [67.1 – 71.1] |
| Duration of infertility (months) | 39.2 ± 14.4  [34.6 – 43.9] | 42.8 ± 16.2  [40.1 – 45.5] |
| Caucasian (%) | 49/60 (81.7%) | 166/196 (84.7%) |
| Causes of infertility (%) |  |  |
| - Male subfertility | 36/60 (60.0%) | 120/196 (61.2%) |
| - Idiopathic | 11/60 (18.3%) | 30/196 (15.3%) |
| - Tubal factor infertility | 11/60 (18.3%) | 37/196 (18.9%) |
| - Polycystic ovarian syndrome | 2/60 (3.3%) | 19/196 (9.7%) |
| - others | 3/60 (5.0%) | 10/196 (5.1%) |
| Number of patients with pre-existing diseases (%) |  |  |
| - No pre-existing diseases | 41/60 (68.3%) | 136/196 (69.4%) |
| - Hypothyroidism | 13/60 (21.6%) | 35/196 (17.9%) |
| - Diabetes mellitus type 2 | 0/60 (0%) | 4/196 (2.0%) |
| - Others (e.g. Asthma; Hypertonus; Migräne) | 6/60 (10.0%) | 21/196 (10.7%) |
| Number of patients with previous surgery (%) |  |  |
| - No prior surgery | 38/60 (63.3%) | 114/196 (58.2%) |
| - Laparoscopic surgery | 6/60 (10.0%) | 35/196 (17.9%) |
| - Hysteroscopic surgery | 1/60 (1.6%) | 6/196 (3.1%) |
| - Hysteroscopy and laparoscopy | 5/60 (8.3%) | 15/196 (7.7%) |
| - Cesarean section | 2/60 (3.3%) | 12/196 (6.1%) |
| - Currettage | 5/60 (8.3%) | 6/196 (3.1%) |
| - Conisation | 3/60 (5.0%) | 8/196 (4.1%) |
| Number of patients with regular menstrual cycle (%) | 48/60 (80.0%) | 154/196 (78.6%) |
| Number of patients smoking (%) | 9/60 (15.0%) | 41/196 (20.9%) |
| Number of patients with polycystic ovarian syndrome (%) | 6/60 (10.0%) | 28/196 (14.3%) |
| Number of patients with endometriosis (%) | 1/60 (1.7%) | 14/196 (7.1%) |
| Number of patients with previous ectopic pregnancy (%) | 3/60 (5.0%) | 13/196 (6.6%) |
| Patient with no previous delivery (%) | 40/60 (66.7%) | 98/196 (50.0%) |
| Number of patients with previous miscarriage (%) | 14/60 (23.3%) | 43/196 (21.9%) |
| Number of patients with no previous embryo transfer before inclusion into analysis (%) | 20/60 (33.3%) | 60/196 (30.6%) |
| Number of 2PNs cryopreserved at prior ovum pick-up | 7.93 ± 4.86  [6.60 – 9.25] | 8.11 ± 5.38  [7.31 – 8.92] |
| Number of embryos transferred | 1.33 ± 0.475  [1.21 – 1.46] | 1.30 ± 0.458  [1.23 – 1.36] |
| Proportion of patients with top quality embryo transfer (%) | 48/60 (80.0%) | 113/196 (57.7%) |
| Endometrial thickness (mm) at embryo transfer | 9.54 ± 6.89  [7.68 – 11.4] | 8.85 ± 1.91  [8.57 – 9.12] |
| Pelvic inflammatory disease | 2/60 (3.3%) | 9/196 (4.6%) |
| Add ons |  |  |
| - Embryo glue | 4/60 (6.7%) | 25/196 (12.8%) |
| - Assisted hatching | 0/60 (0.0%) | 5/196 (2.6%) |
| Dydrogesterone at ET (ng/ml) (n =186) | 1.41 ± 0.928  [1.14 – 1.69] | 1.39 ± 1.00  [1.23 – 1.56] |
| Dihydrogesterone at ET (ng/ml) (n =186) | 38.6 ± 21.9  [32.0 – 45.2] | 37.1 ± 23.1  [33.3 – 41.0] |
| Progesterone at ET (ng/ml) (n = 88) | 0.140 ± 0.125  [0.0859 – 0.194] | 0.108 ± 0.0908  [0.0850 – 0.130] |
| Estradiol at ET (pg/ml) (n = 216) | 234 ± 112  [203 – 264] | 206 ± 87.3  [193 – 220] |
| Dydrogesterone at hCG (ng/ml) (n = 154) | 1.67 ± 0.889  [1.40 – 1.95] | 1.66 ± 1.19  [1.43 – 1.88] |
| Dihydrogesterone at ET (ng/ml) (n = 155) | 48.7 ± 25.7  [40.7 – 56.7] | 45.3 ± 27.3  [40.2 – 50.3] |
| Progesterone at hCG (ng/ml) (n = 96) | 3.45 ± 7.35  [1.07 – 5.83] | 0.301 ± 1.38  [-0.0645 – 0.667] |
| Estradiol at hCG (pg/ml) (n = 189) | 447 ± 291  [363 – 530] | 300 ± 172  [271 – 329] |

Depicted are mean and SD or numbers and proportions, as appropriate. hCG: Human chorionic gonadotropin; 2PN: 2 pronuclei embryo; FET: frozen embryo transfer.

**Supplementary Table S3: Probability values of the prediction model including dydrogesterone values and outcome of embryo transfer in women of the low-likelihood group.**

| Patient ID | Probabilities | Implantation | Clinical pregnancy | Live birth |
| --- | --- | --- | --- | --- |
| 30 | 0.05182537 | no | no | no |
| 17 | 0.00679708 | no | no | no |
| 8 | 0.05308366 | no | no | no |
| 45 | 0.0653825 | no | no | no |
| 13 | 0.19150124 | no | no | no |
| 1 | 6.76E-07 | no | no | no |
| 286 | 0.16684814 | yes | no | no |
| 299 | 0.23361169 | no | no | no |
| 318 | 2.22E-16 | no | no | no |
| 195 | 0.07786268 | no | no | no |
| 180 | 2.22E-16 | no | no | no |
| 172 | 2.22E-16 | no | no | no |
| 215 | 0.24866496 | yes | no | no |
| 241 | 0.01852133 | no | no | no |
| 101 | 1.98E-10 | no | no | no |
| 110 | 0.14736091 | no | no | no |
| 147 | 0.05543352 | no | no | no |
| 153 | 2.22E-16 | no | no | no |
| 156 | 0.00011615 | no | no | no |
| 158 | 0.16541719 | no | no | no |
| 160 | 3.50E-08 | no | no | no |
| 161 | 0.08326144 | no | no | no |
| 164 | 1.78E-10 | no | no | no |
| 78 | 1.08E-08 | no | no | no |

**Supplementary Table S4: Probability values and outcome of embryo transfer in women of the low-likelihood group without dydrogesterone values.**

| Patient ID | Implantation | Clinical Pregnancy | Live birth | Probability value |
| --- | --- | --- | --- | --- |
| 17 | no | no | no | 0.01859064 |
| 45 | no | no | no | 0.22574639 |
| 13 | no | no | no | 0.09577143 |
| 1 | no | no | no | 0.00119624 |
| 37 | yes | no | no | 0.21457891 |
| 318 | no | no | no | 2.22E-16 |
| 195 | no | no | no | 0.01190038 |
| 172 | no | no | no | 2.22E-16 |
| 241 | no | no | no | 0.12726903 |
| 101 | no | no | no | 5.45E-05 |
| 110 | no | no | no | 0.10336789 |
| 147 | no | no | no | 0.216089 |
| 153 | no | no | no | 1.04E-08 |
| 156 | no | no | no | 0.07436263 |
| 160 | no | no | no | 0.00026205 |
| 164 | no | no | no | 9.28E-06 |
| 78 | no | no | no | 0.00011887 |

**Supplementary Table S5: Most prevalent taxa identified as indicators^#^ for the low-likelihood group of women undergoing IVF-treatment identified without dydrogesterone as predictor.**

| Taxon | A* | B** | p-value |
| --- | --- | --- | --- |
| *Ureaplasma parvum* | 0.96291 | 1 | ** |
| *Lactobacillus coleohominis* | 0.72616 | 0.29412 | * |
| *Chlamydia trachomatis* | 0.99914 | 0.17647 | * |
| Nostocaceae EU753646 g EU753646 s | 0.72447 | 0.23529 | * |
| *Enterococcus faecalis* | 0.70537 | 0.23529 | * |
| *Veillonella montpellierensis* | 0.87193 | 0.17647 | * |
| *Acidovorax wautersii* | 0.82414 | 0.17647 | * |
| *Staphylococcus xylosus* | 0.80834 | 0.17647 | * |

#Indicator species analysis shows the taxa which are significantly associated with low-likelihood of embryo implantation from IVF treatment. A: ratio of total number reads of the respective taxon appearing in low-likelihood group. B: ratio of samples within the low-likelihood group displaying reads of the respective taxon. Statistic: Permutation test within r-package “indicspecies”. *=p<0.05; **=p<0.01.

**
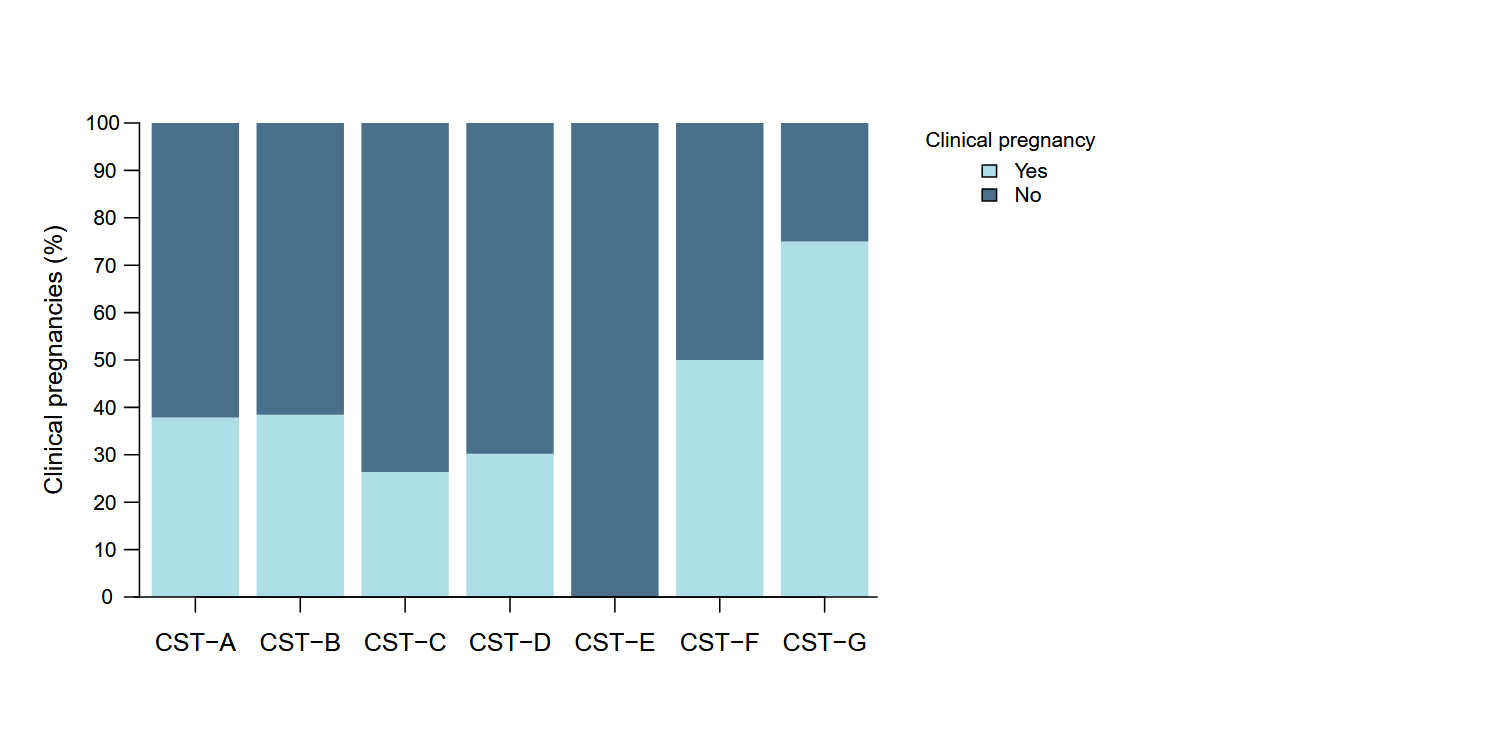
**

C
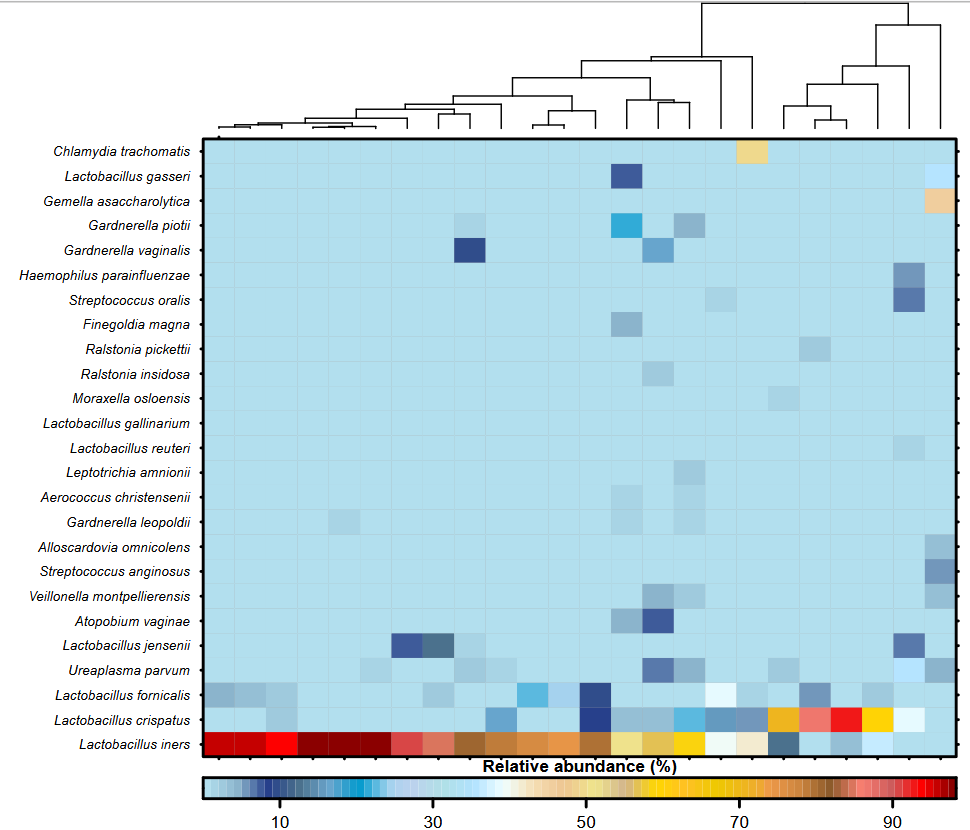


B
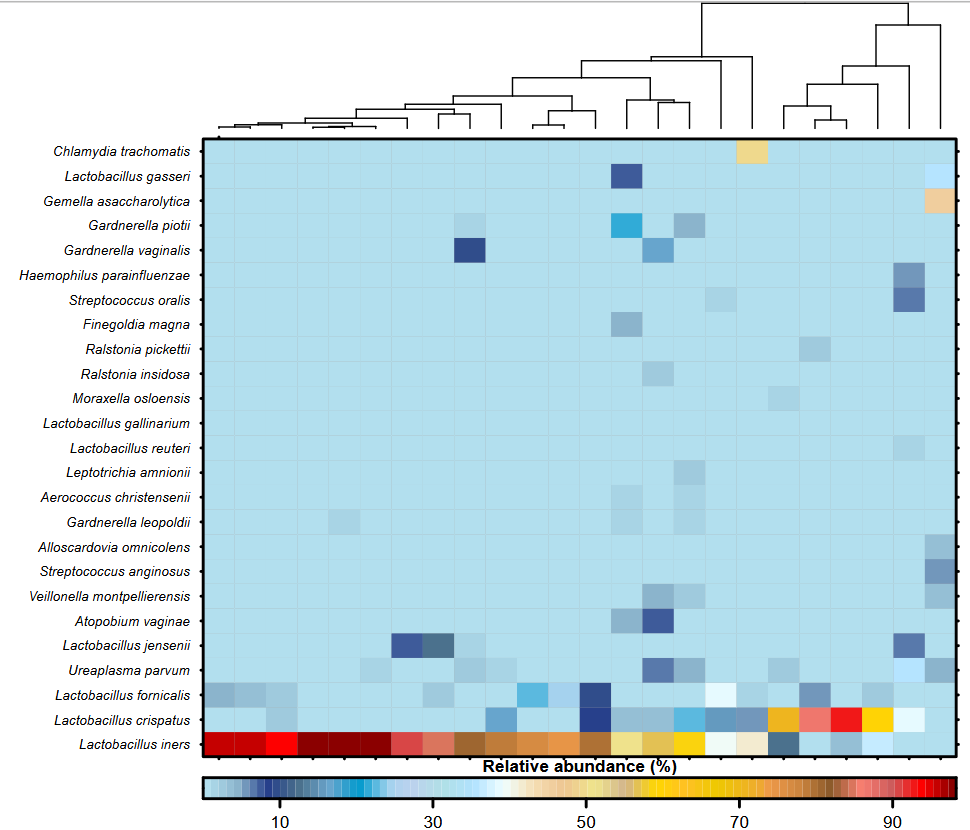


A
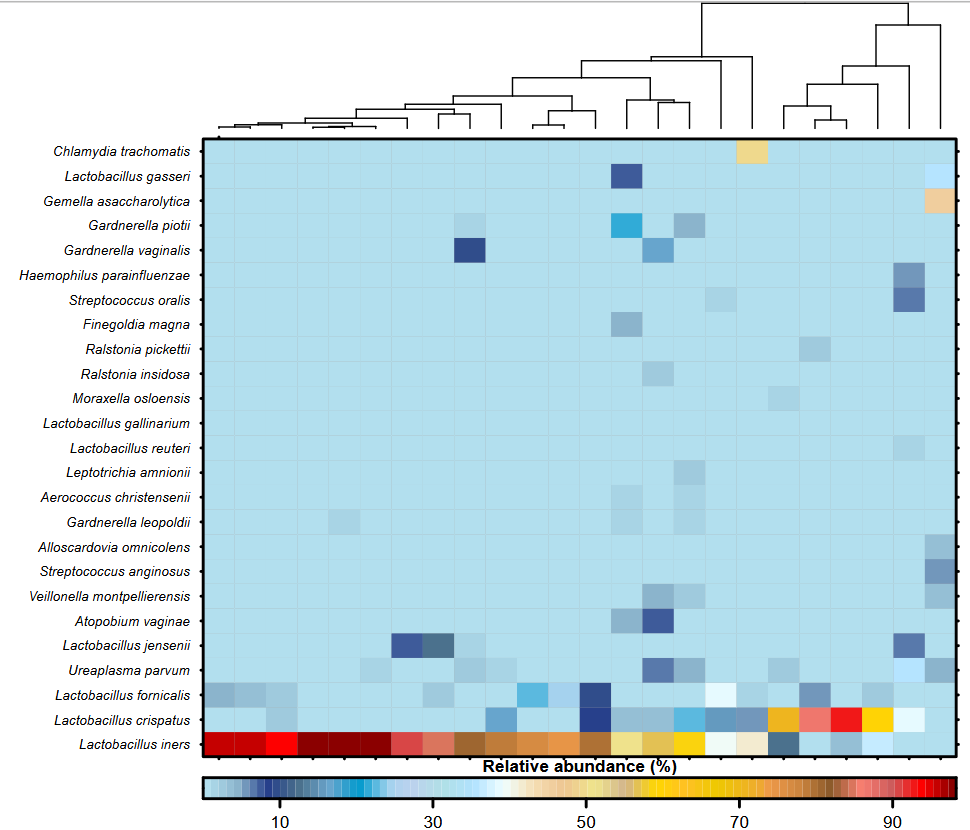


**
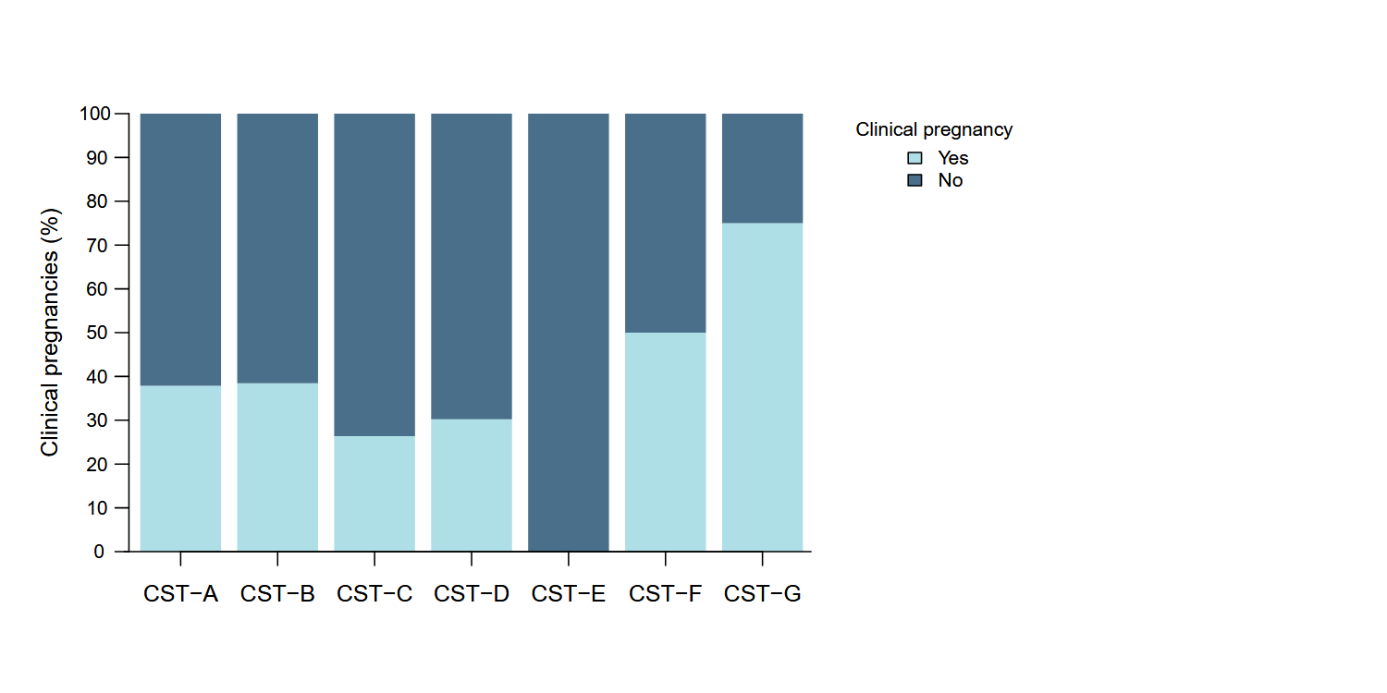

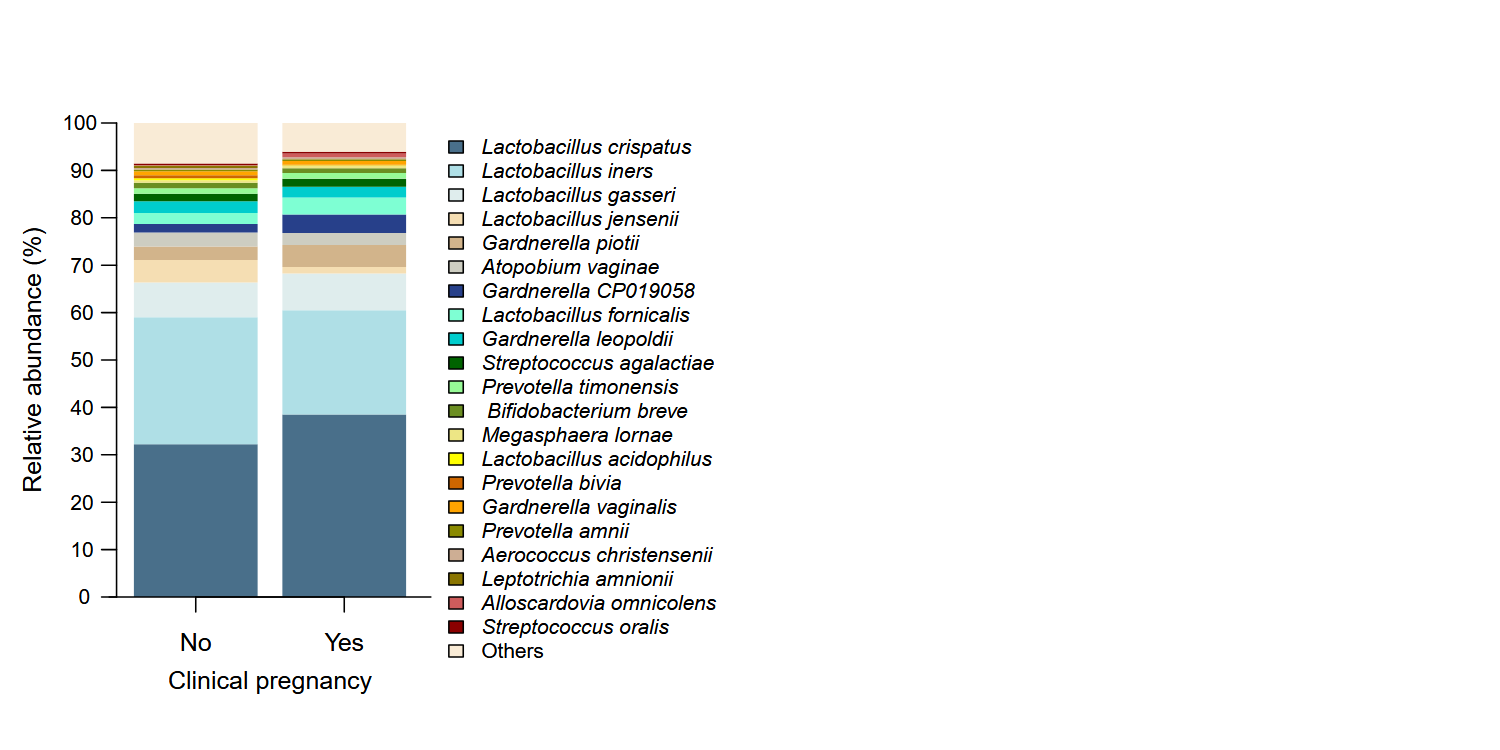
** **
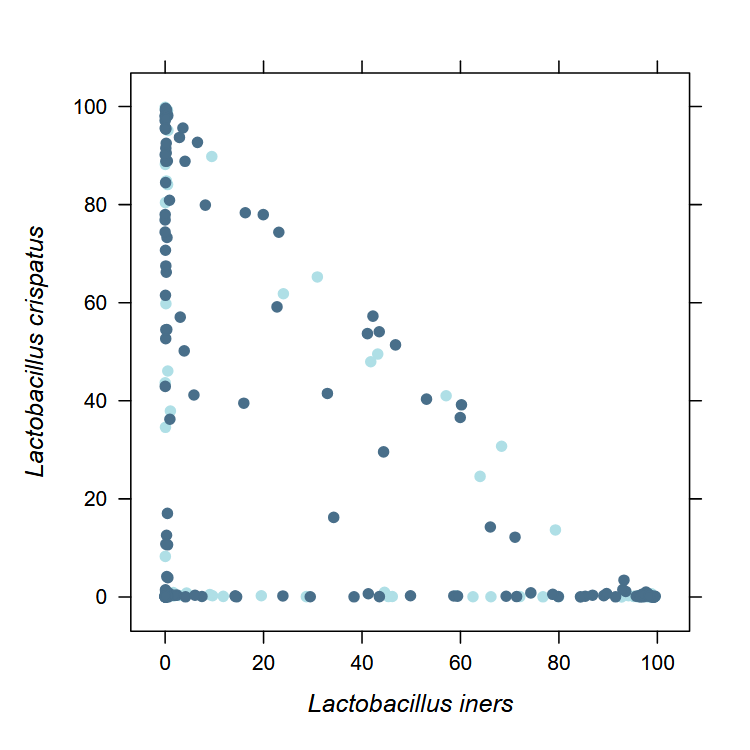
**

F
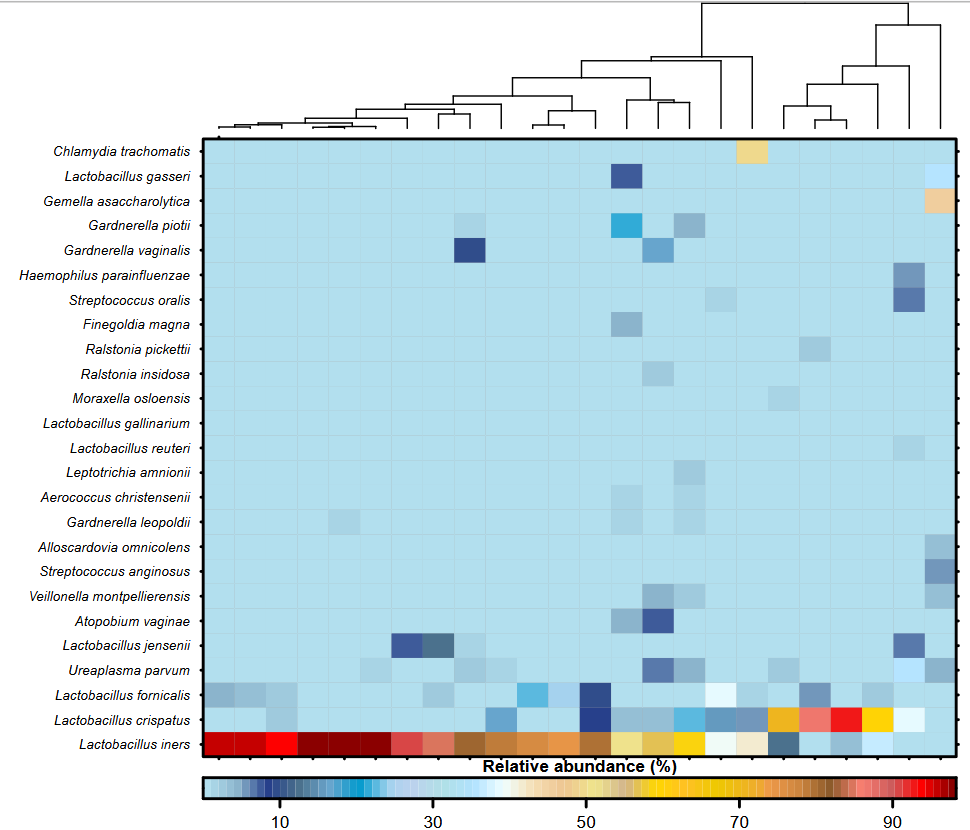


E
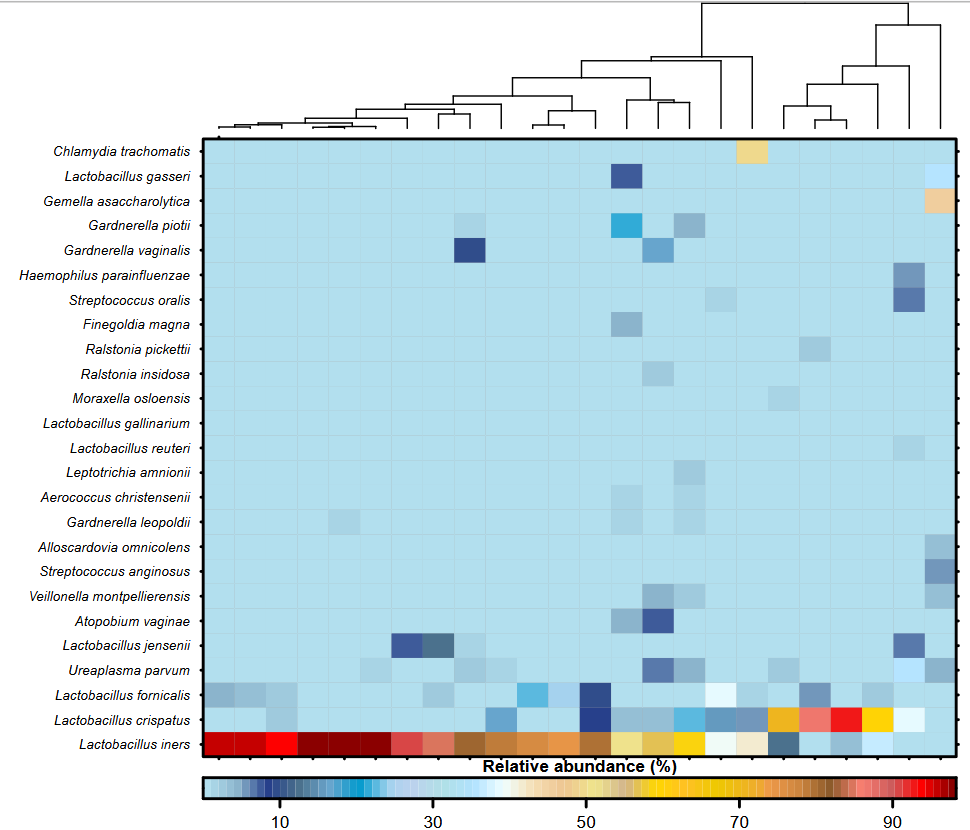


D
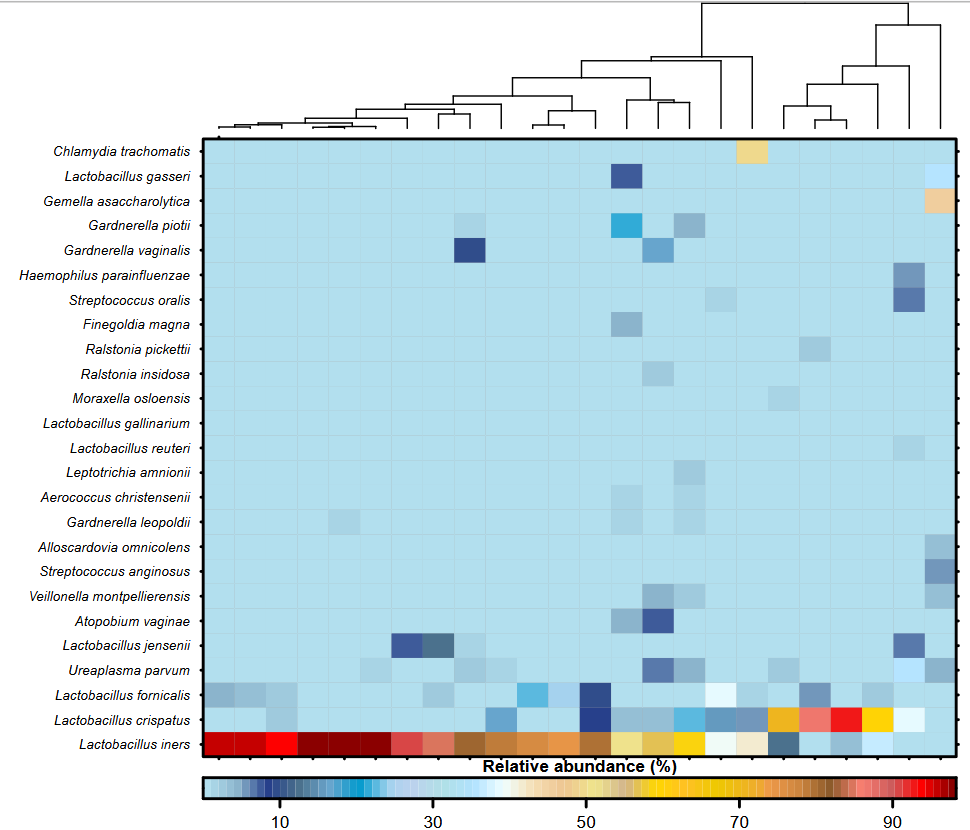


**
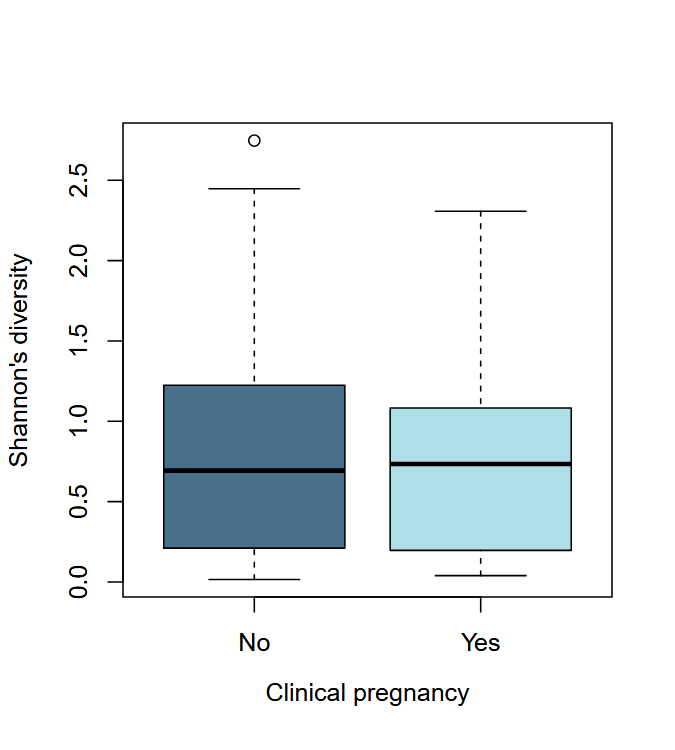

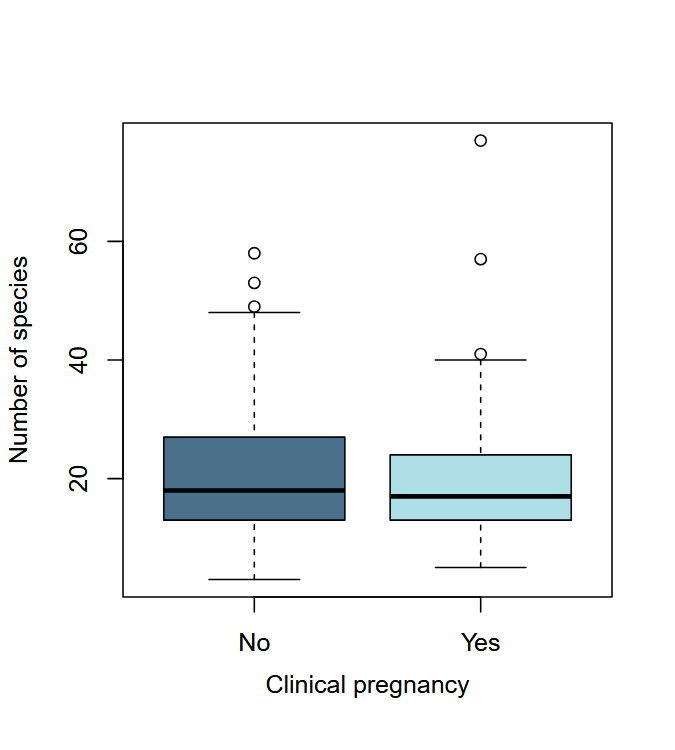

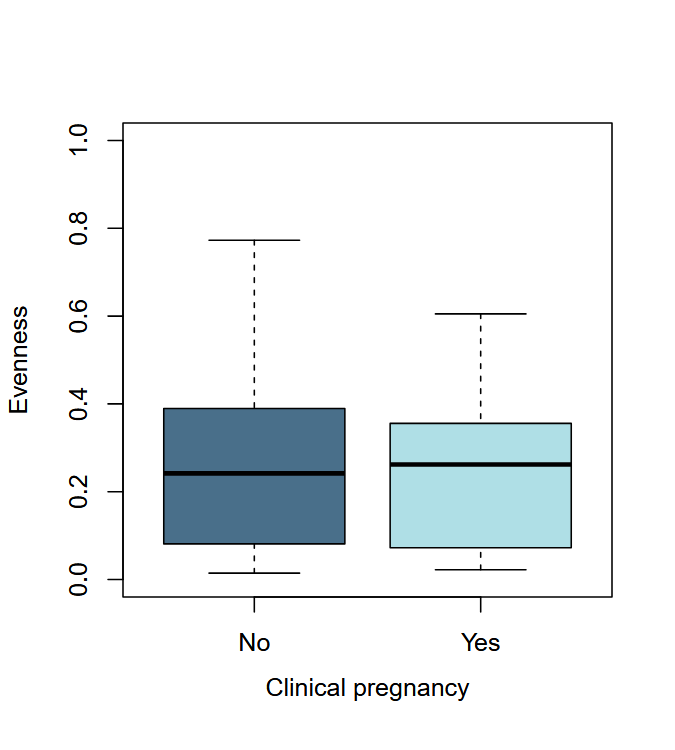
**

**Supplementary Figure S1: Global measures of microbial communities in relation to clinical pregnancy in the cohort.** Variations in clinical pregnancy between CST are statistically not significant (A) and neither relative abundance of major taxa of the data set (B) nor depiction of *L. crispatus* vs*. L iners* relative abundances (C) or alpha diversity measures (D-F), explain occurrence of clinical pregnancy in this cohort. CST: community state type. Statistics: Fisher´s exact test (panel A) and Wilcoxon rank-sum test (panels D-F).


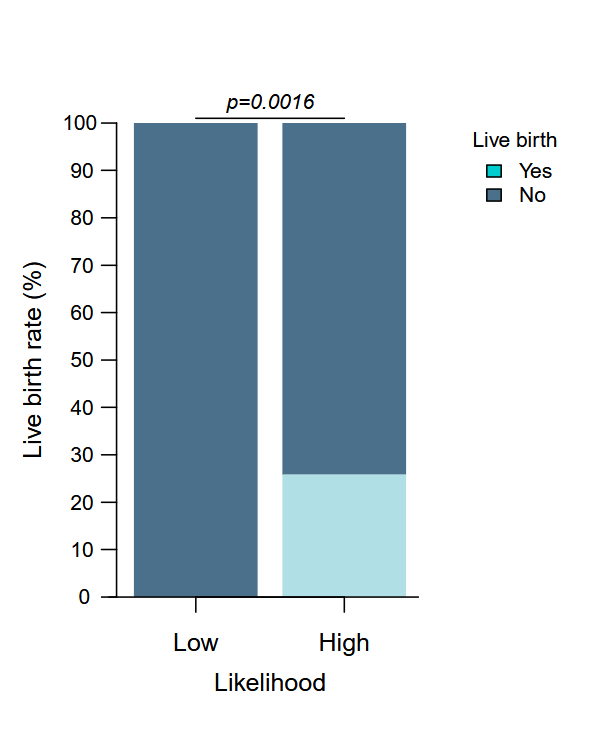

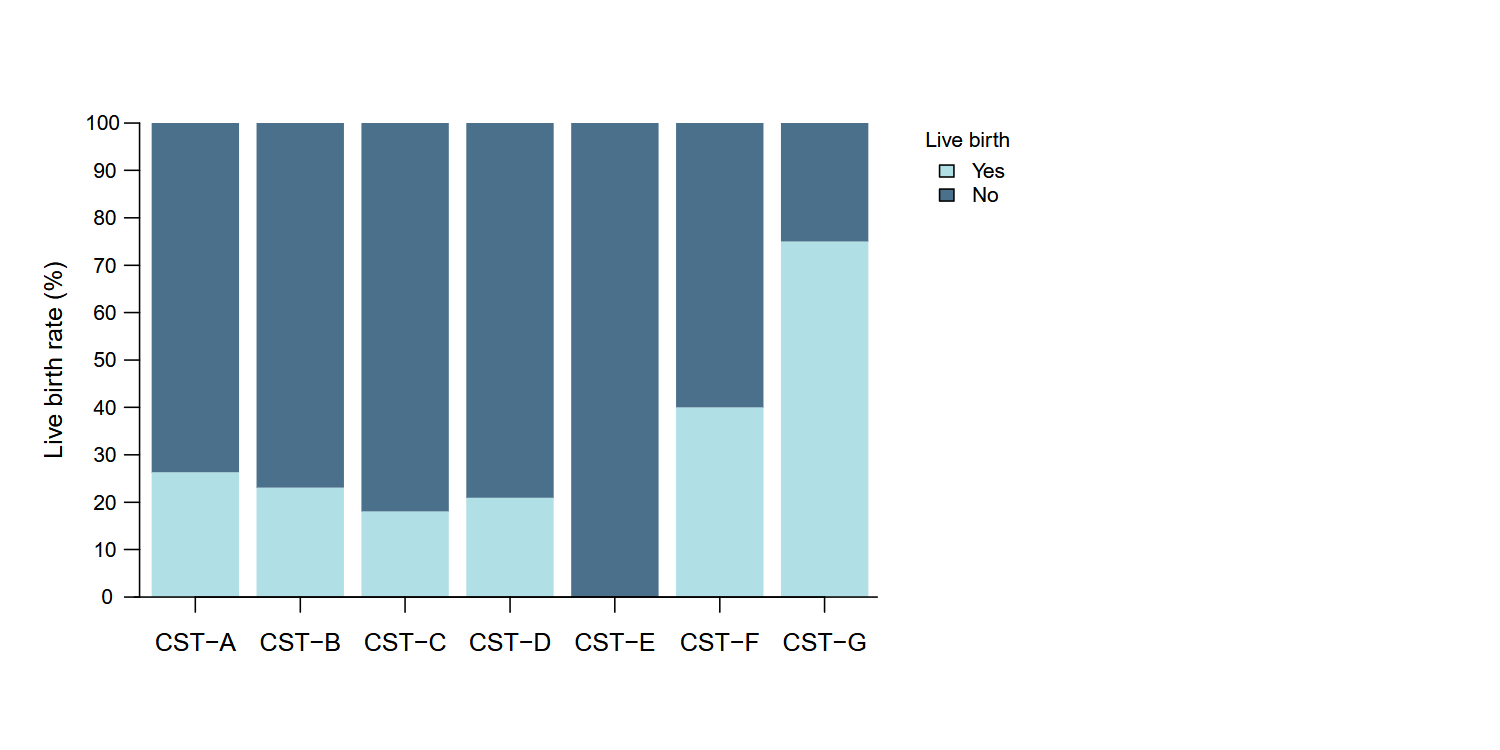

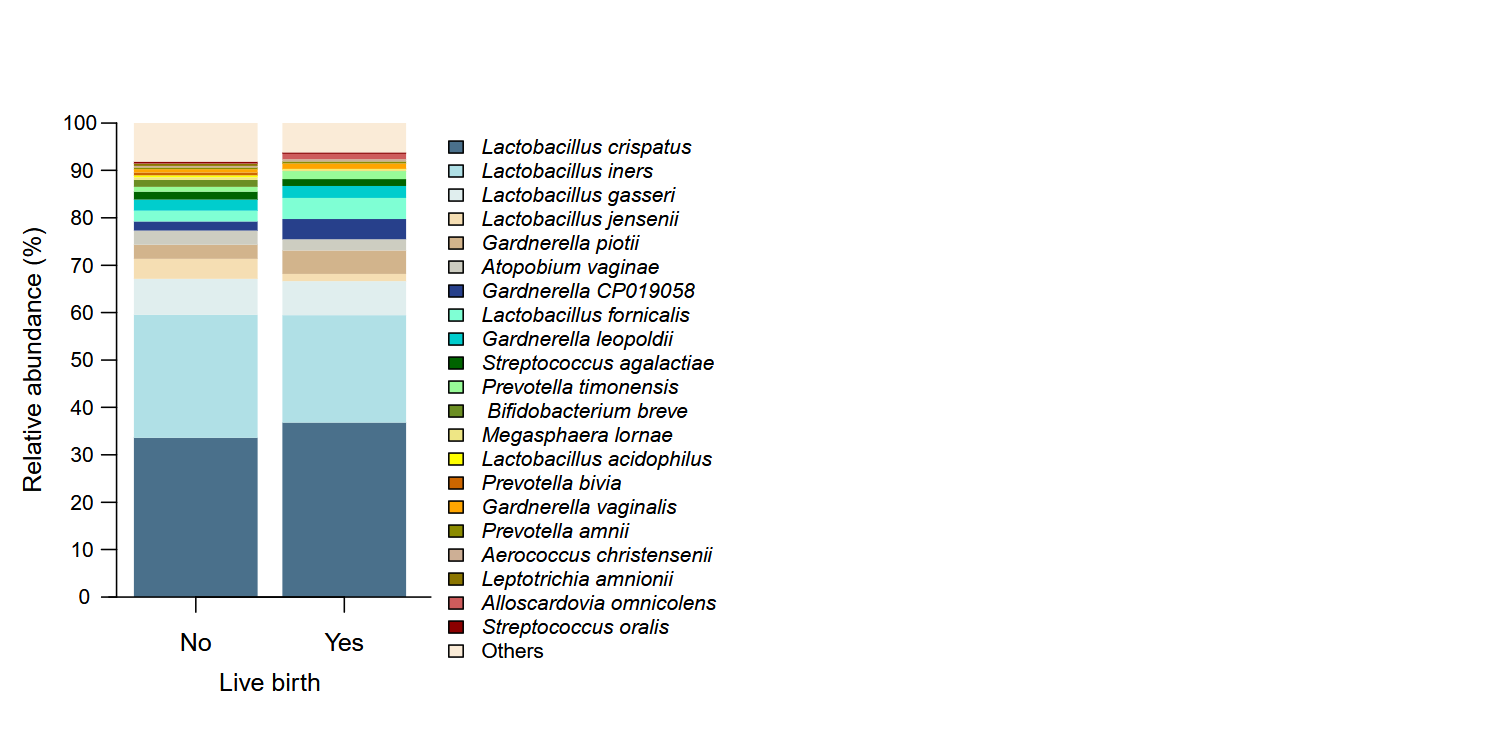
**
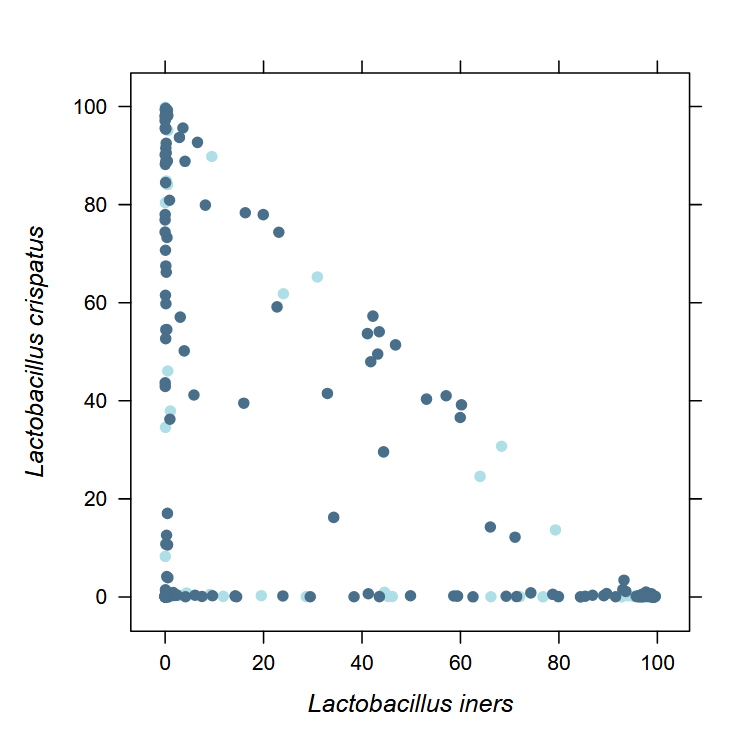
**

F
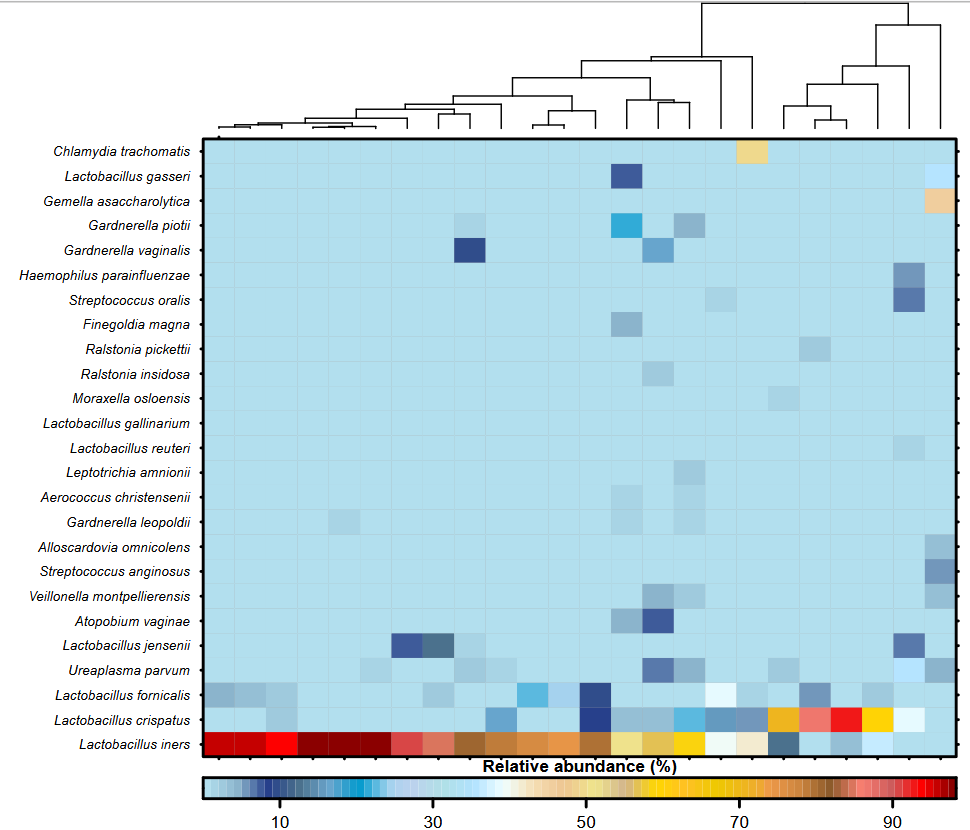


E
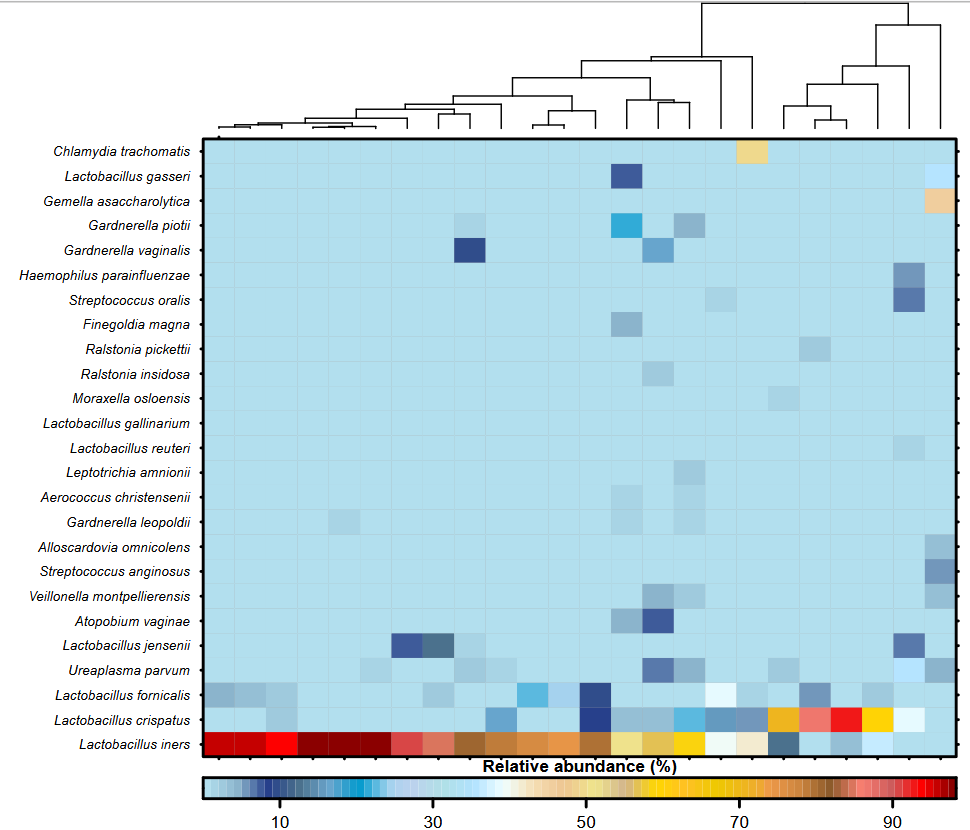


D
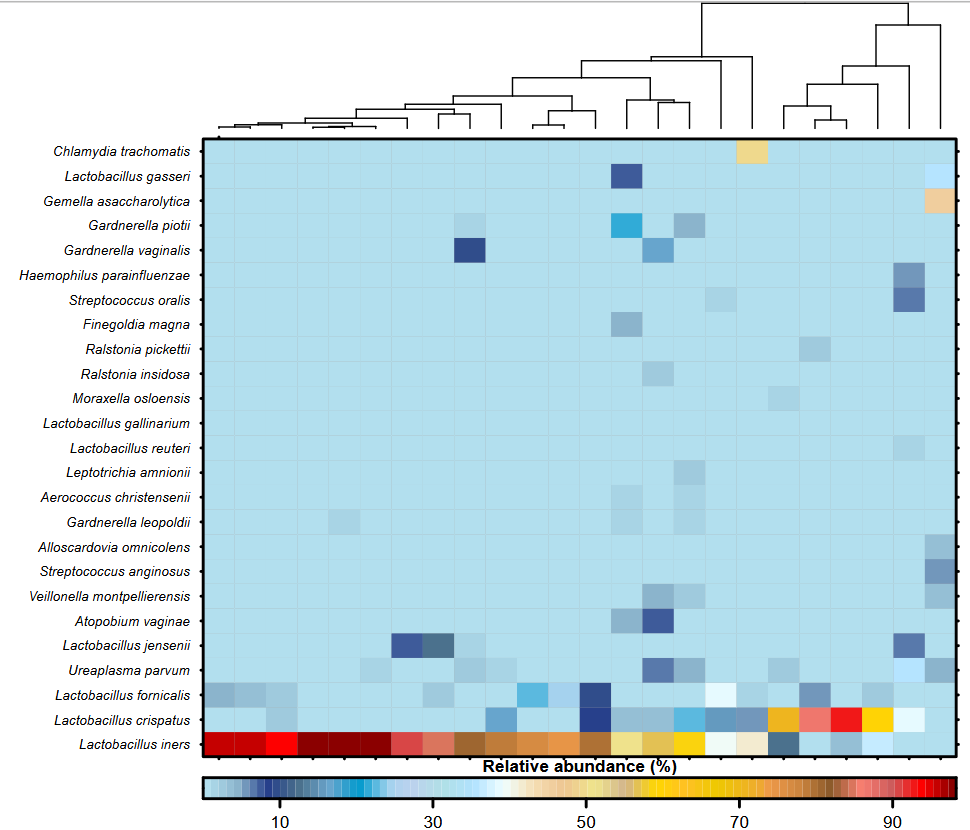


C
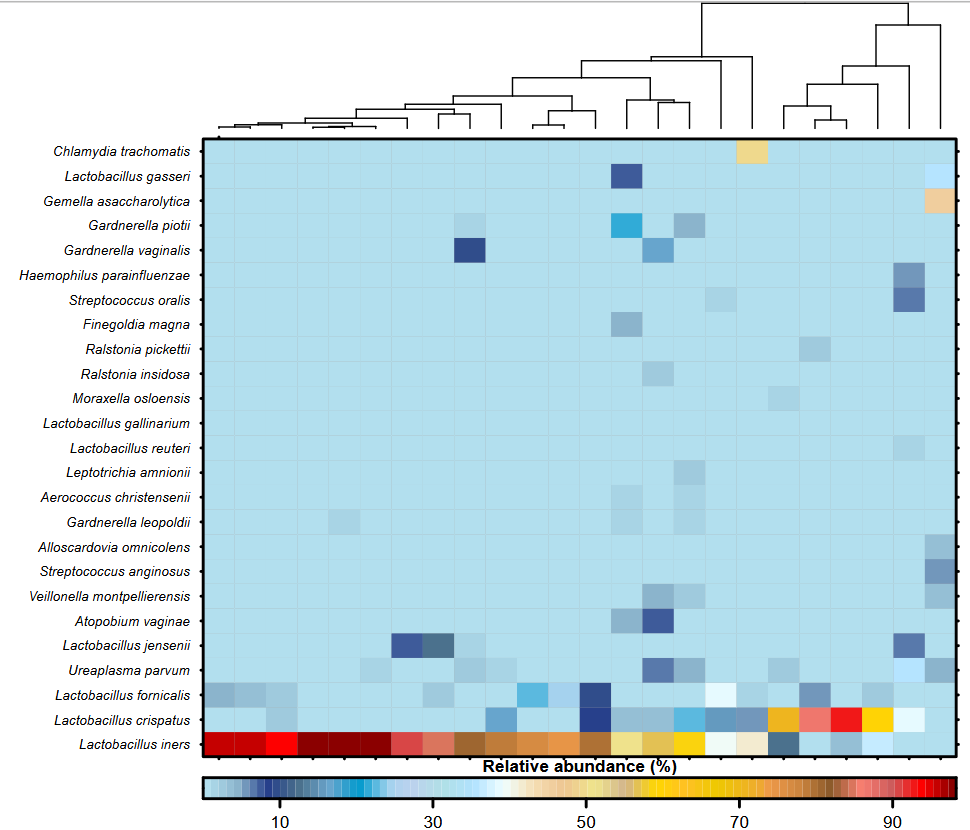


B
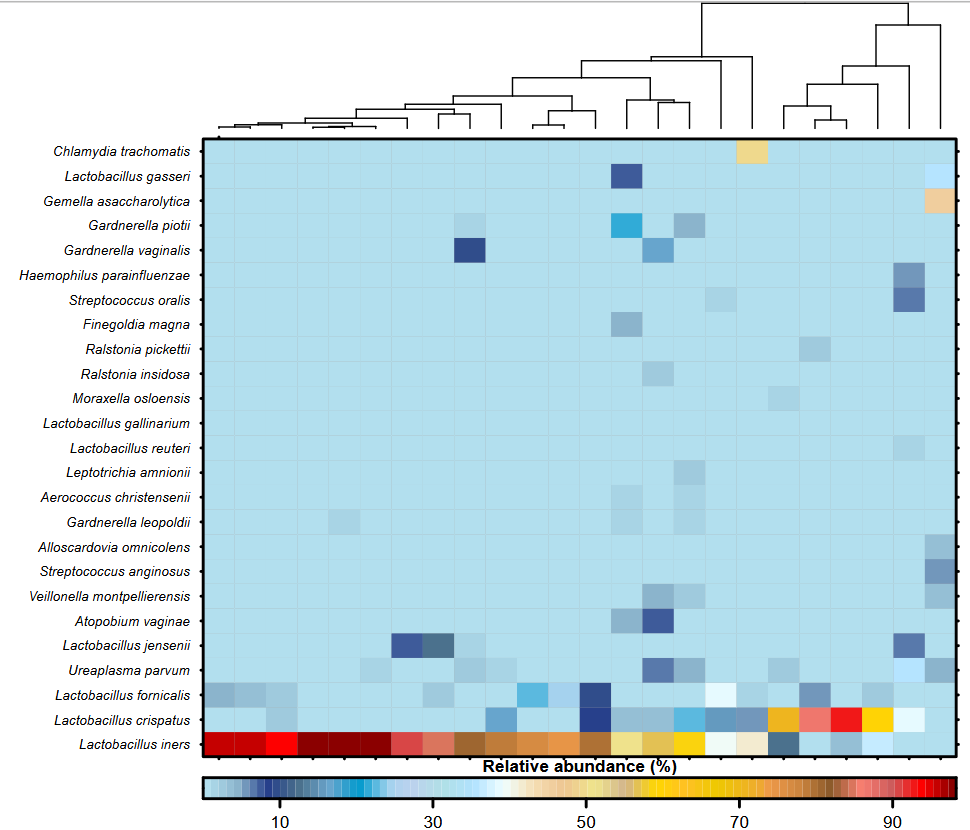


A
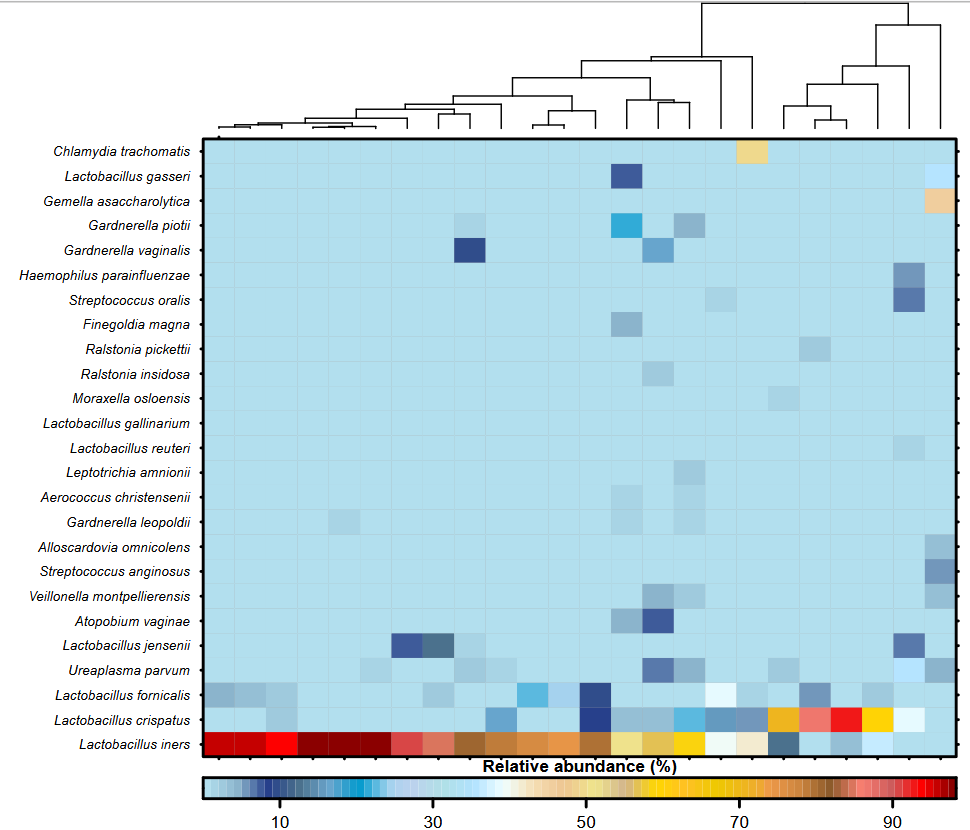


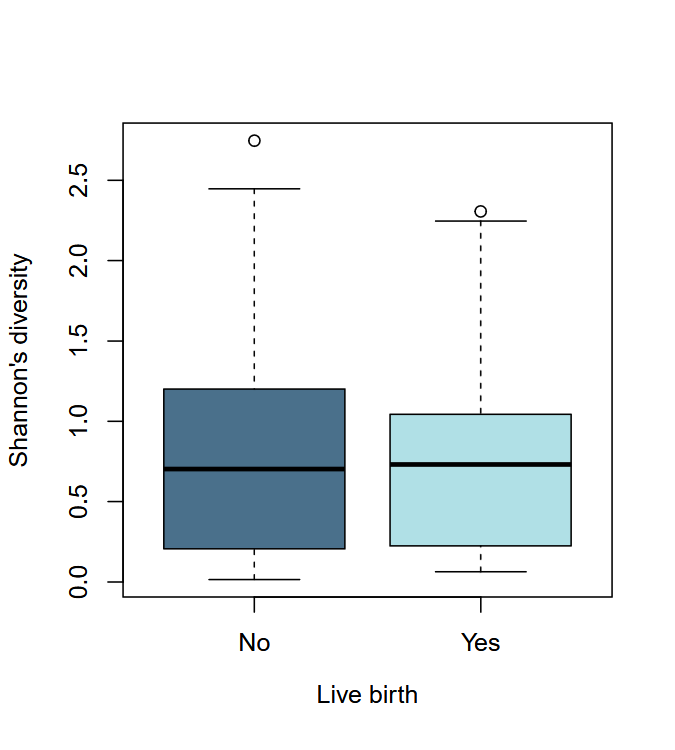

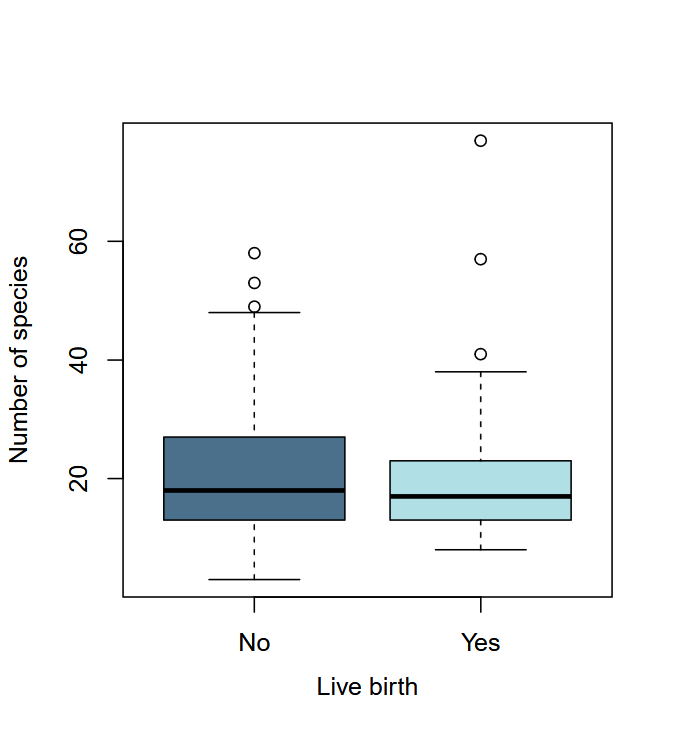

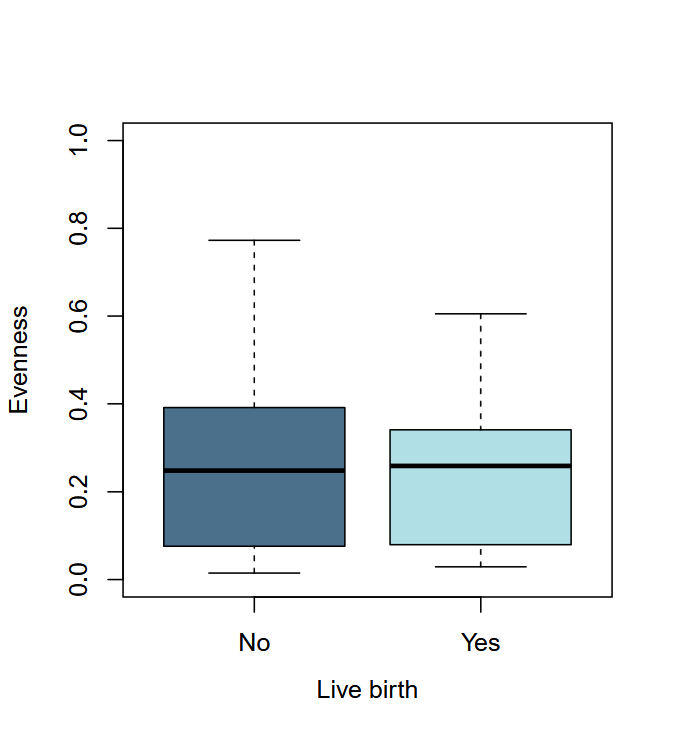


**Supplementary Figure S2: Global measures of microbial communities in relation to giving live birth in the cohort.** Variations in live birth between CST are statistically not significant (A) and neither relative abundance of major taxa of the data set (B) nor depiction of *L. crispatus* vs*. L iners* relative abundances (C) or alpha diversity measures (D-F), explain occurrence of live birth in this cohort. CST: community state type. Statistics: Fisher´s exact test (panel A) and Wilcoxon rank-sum test (panels D-F).

A
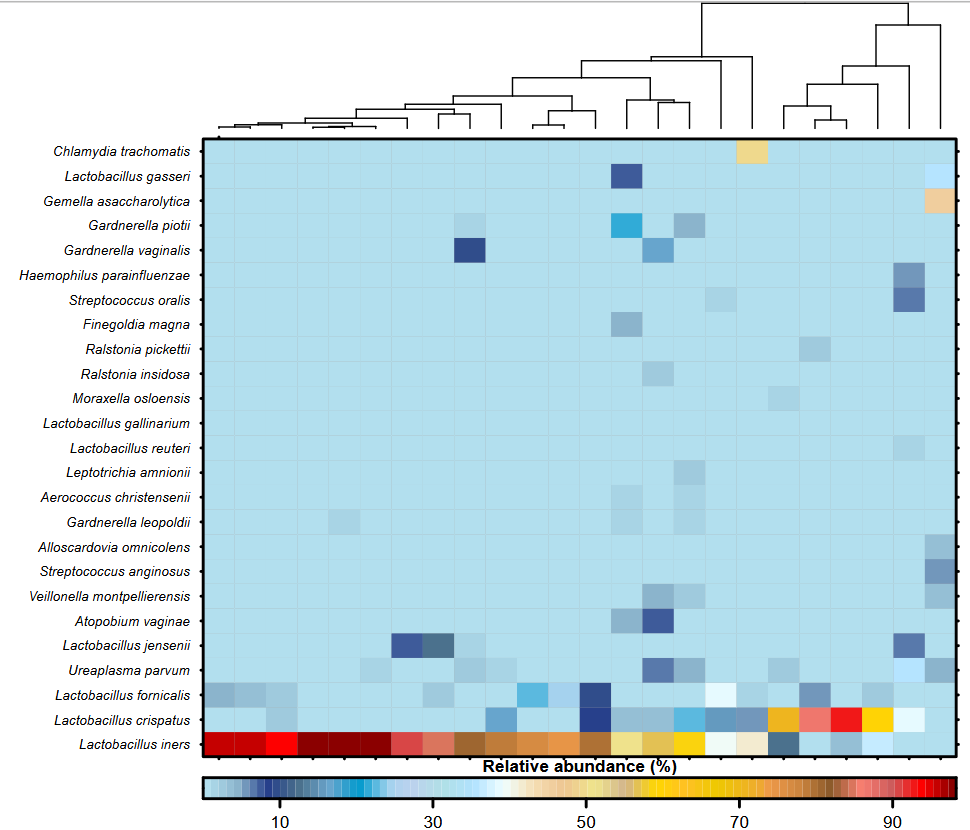


B
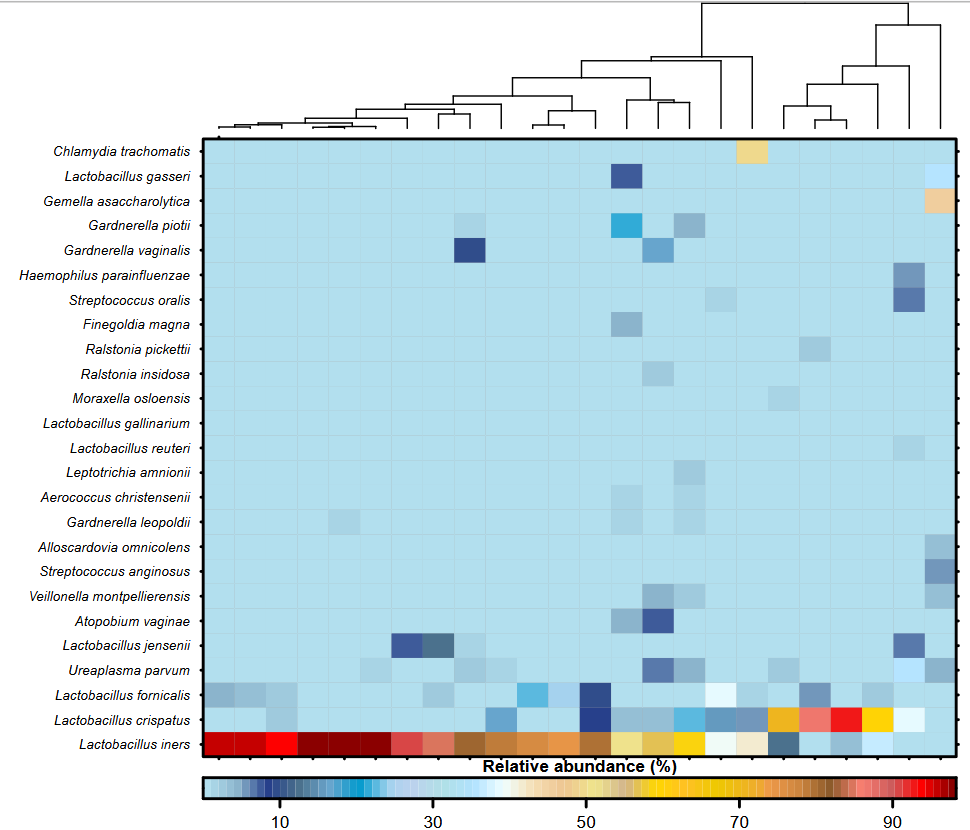


C
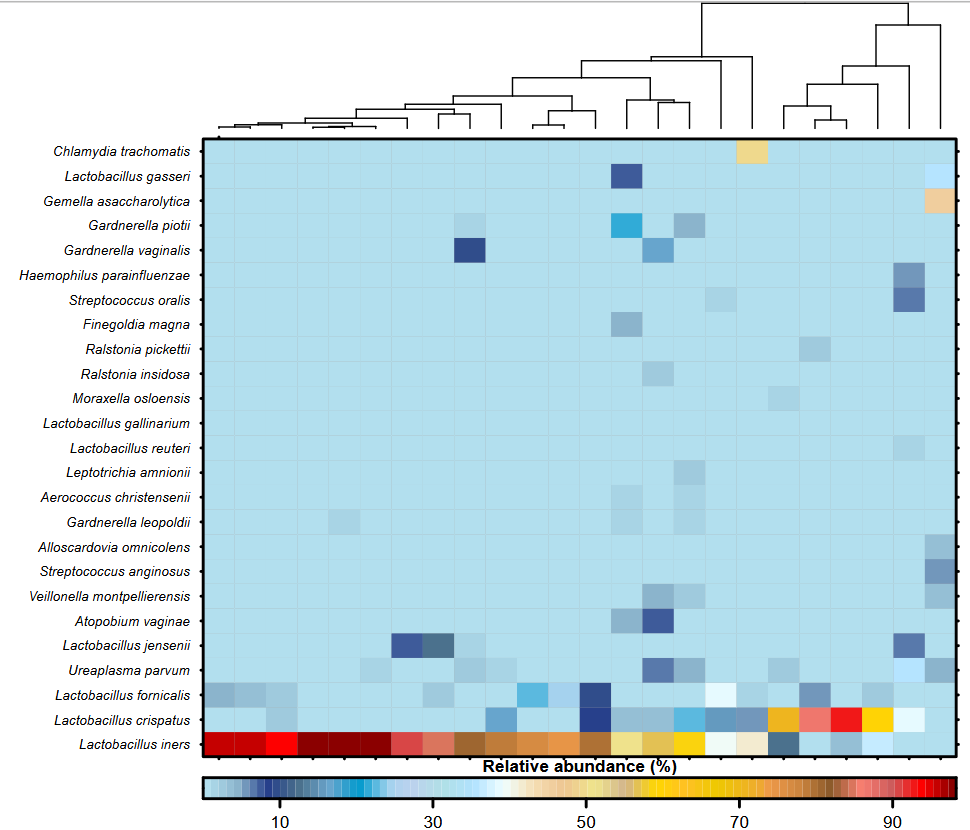


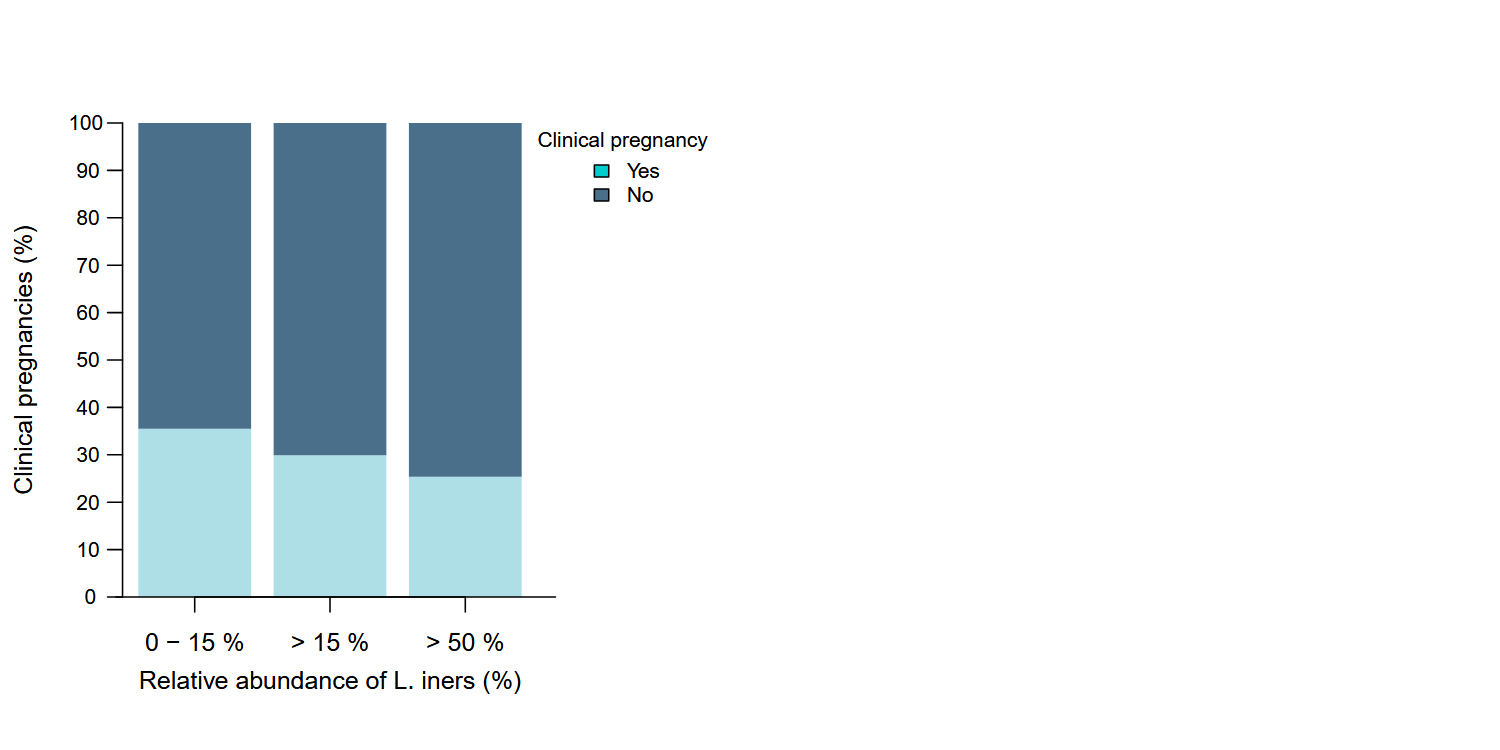

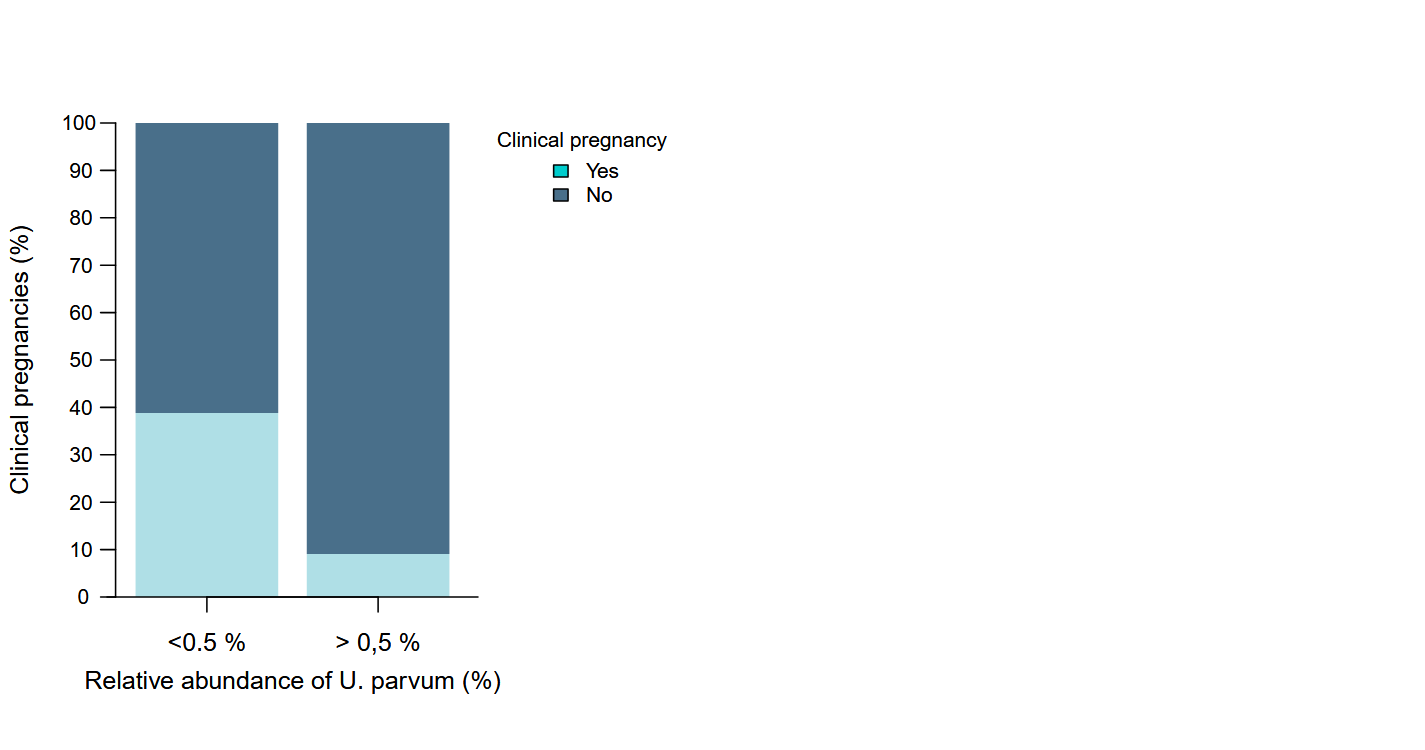

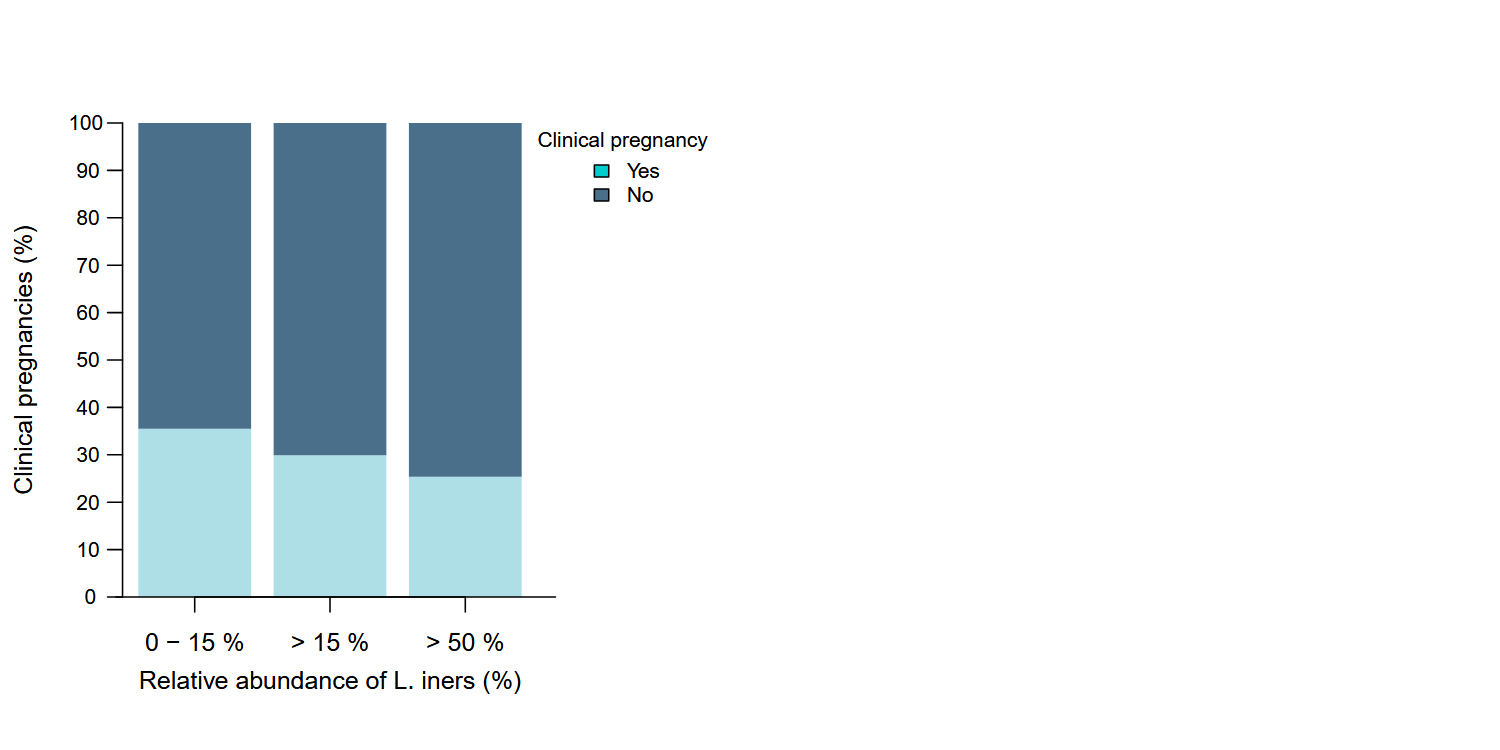


**Supplementary Figure S3: Bacterial predictors´ association to clinical pregnancy in the cohort.** Higher values for *L. iners* yield moderately lower levels of clinical pregnancies (A), while relative abundance of > 0.5 % for *U. parvum* associates with drastically decreased treatment success (B). Contrary, increasing relative abundance of *L. fornicalis* goes along with increased clinical pregnancy rates (C). *L. iners*: *Lactobacillus iners*; *U. parvum*: *Ureaplasma parvum*; *L. fornicalis*: *Lactobacillus fornicalis*.


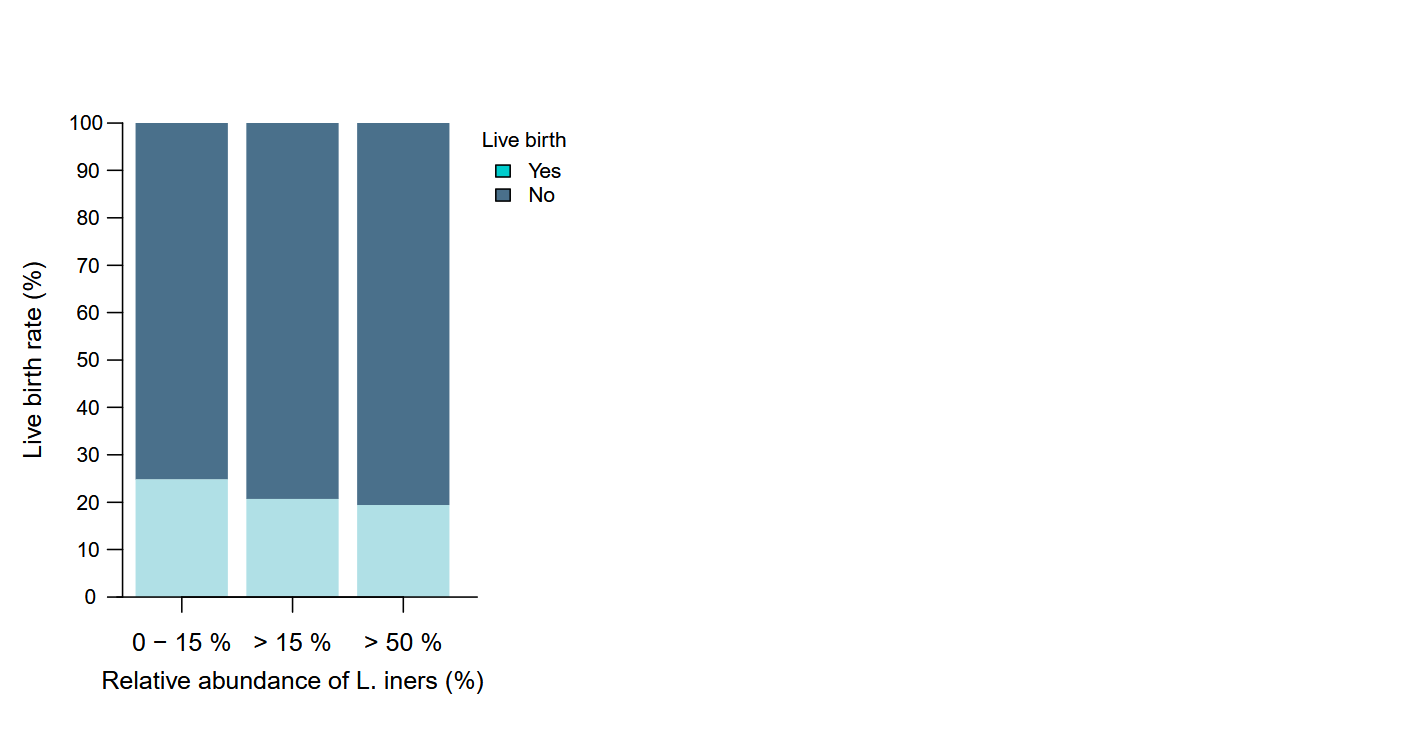

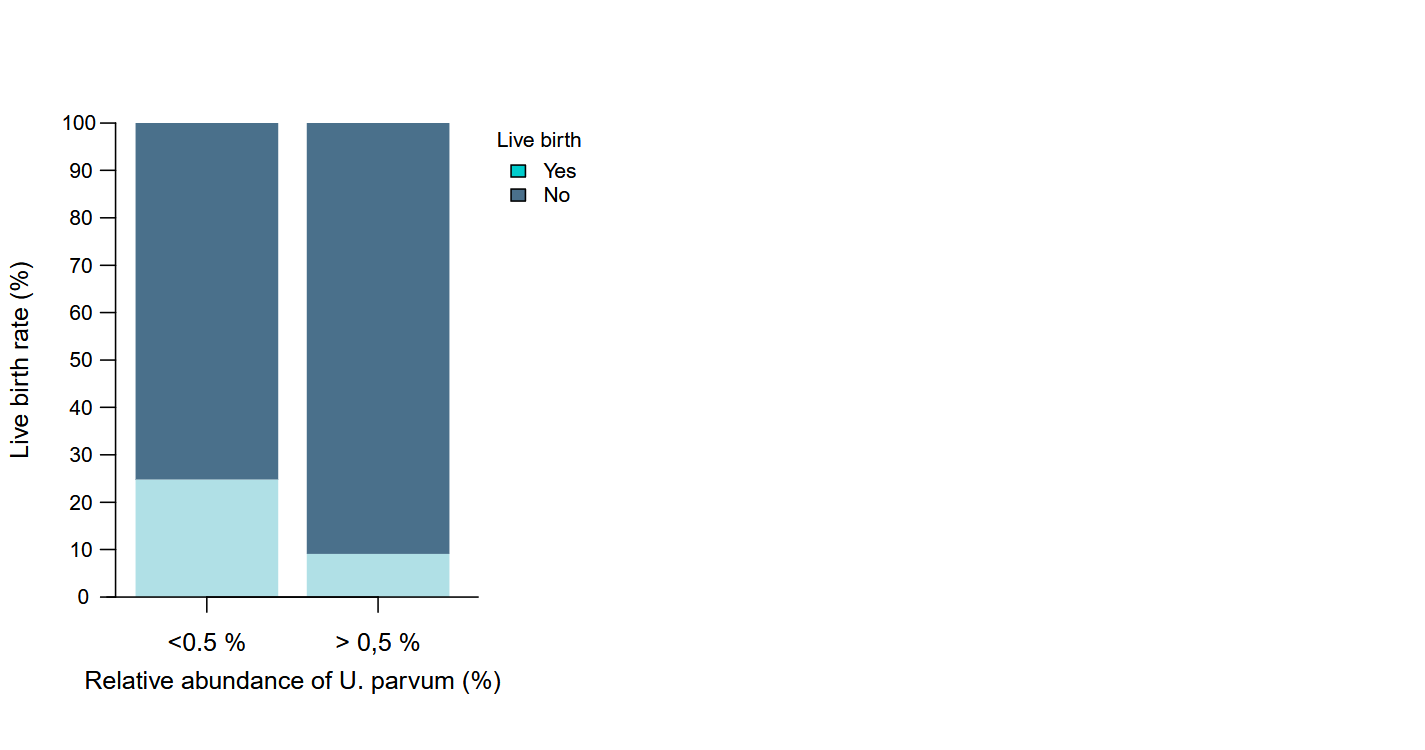

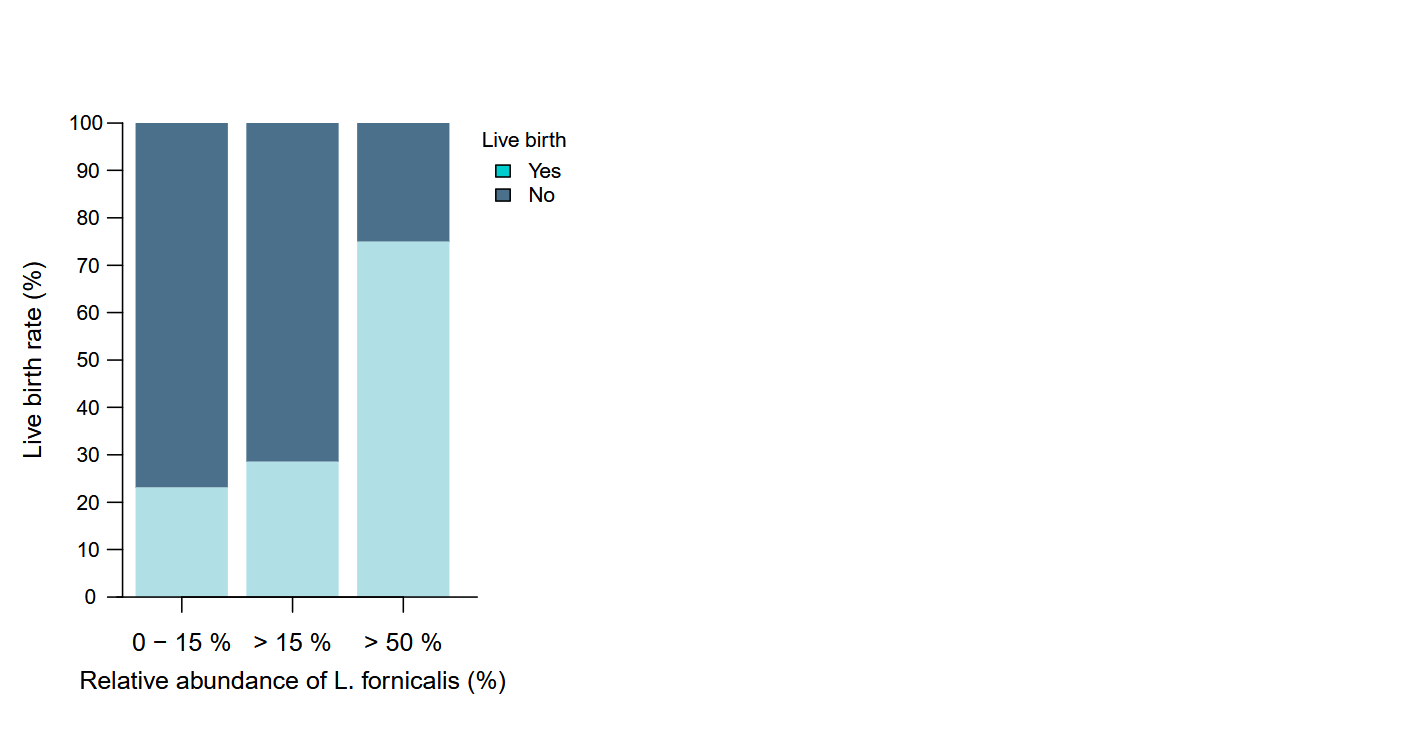


C
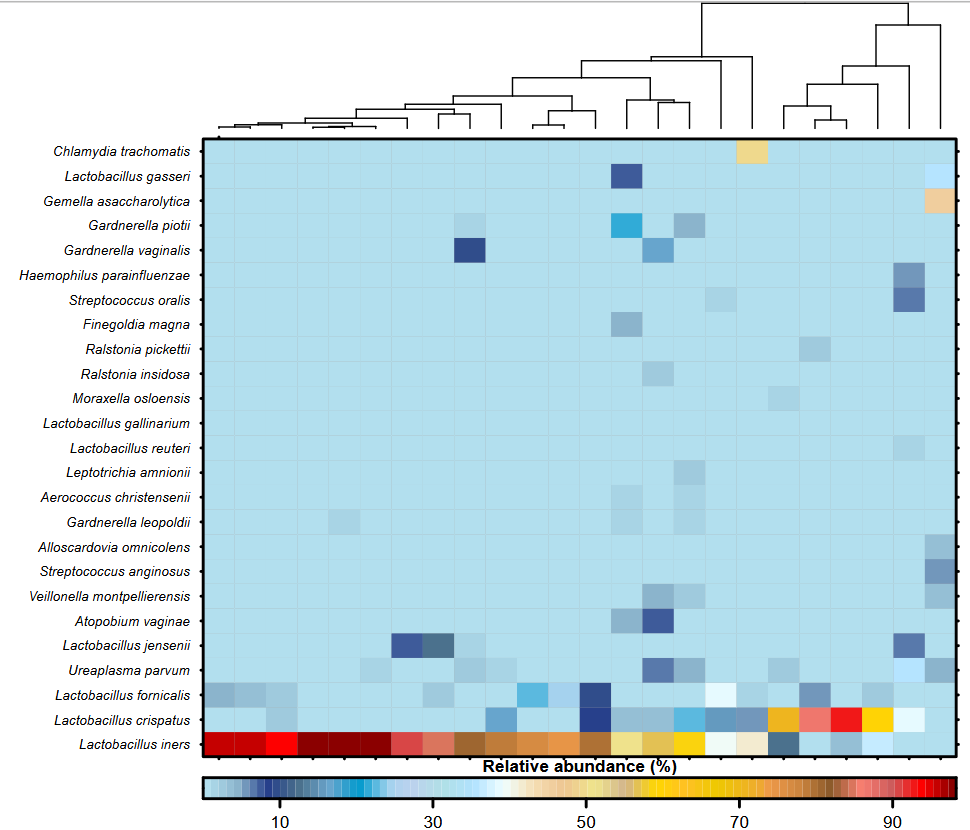


B
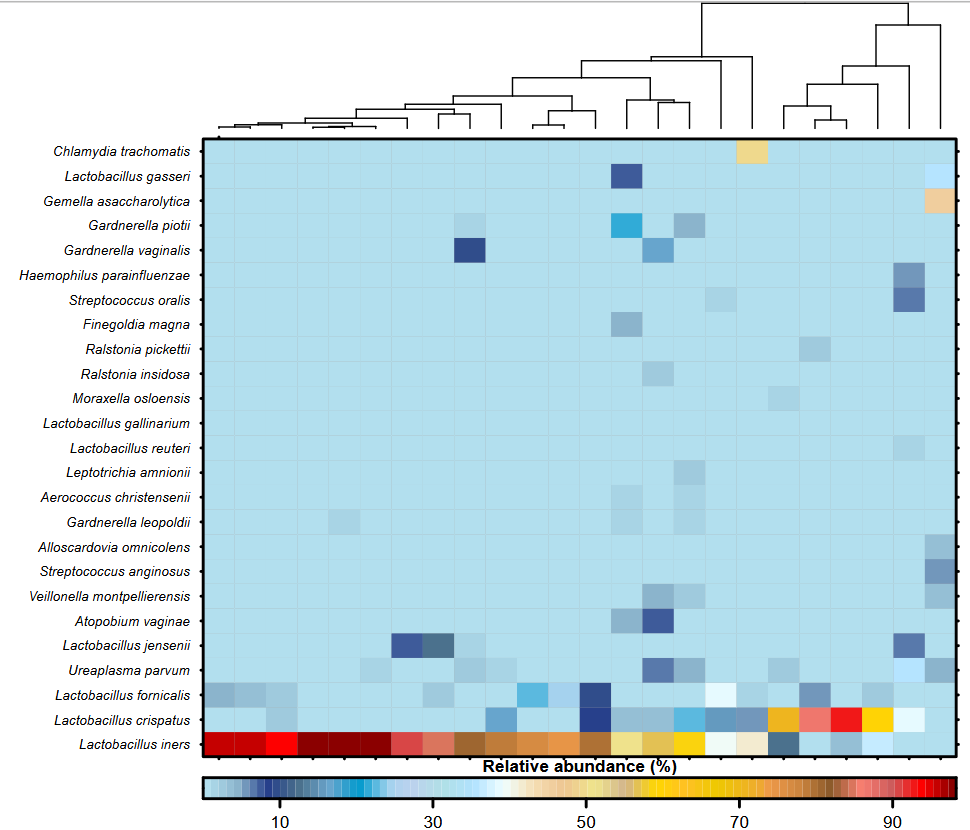


A
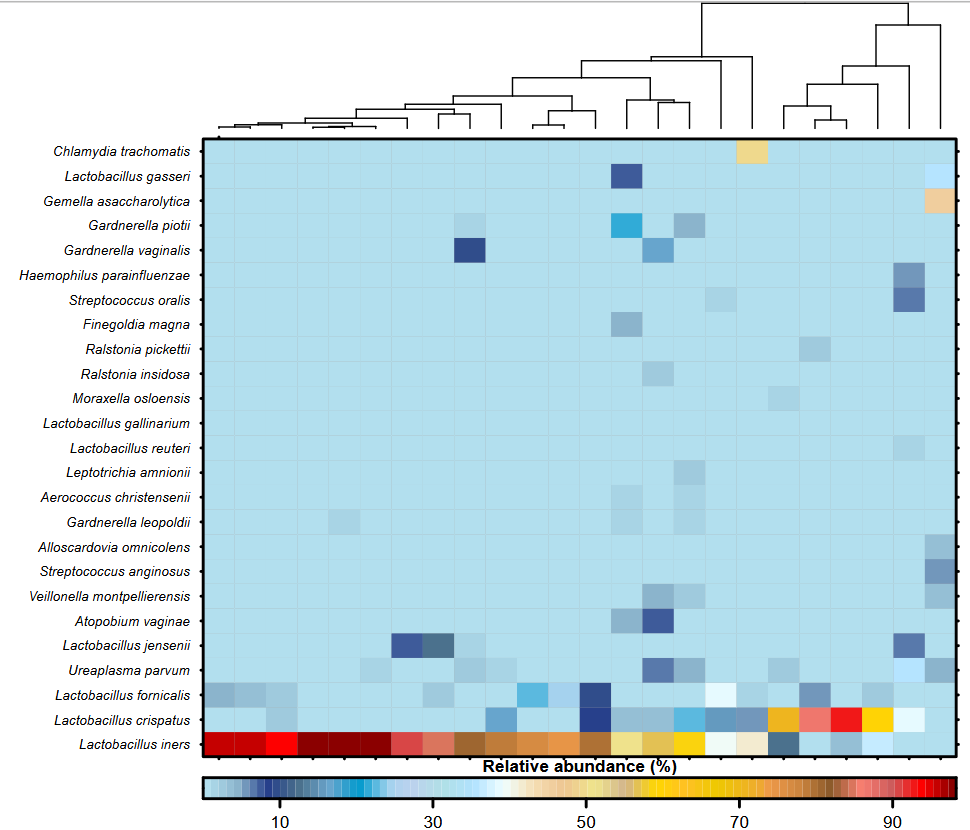


**Supplementary Figure S4: Bacterial predictors´ association to live birth in the cohort.** Higher values for *L. iners* yield moderately lower levels of live birth (A), while relative abundance of > 0.5 % for *U. parvum* associates with drastically decreased treatment success (B). Contrary, increasing relative abundance of *L. fornicalis* goes along with increased live birth (C). *L. iners*: *Lactobacillus iners*; *U. parvum*: *Ureaplasma parvum*; *L. fornicalis*: *Lactobacillus fornicalis*.


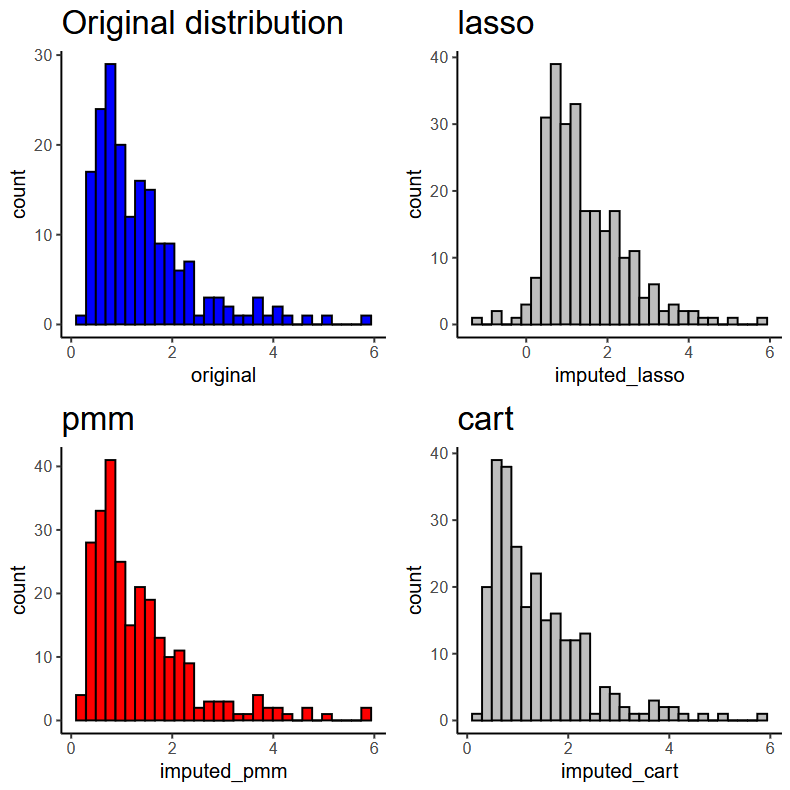


**Supplementary Figure S5: Data distribution of original values for dydrogesterone levels compared to different imputation methods.** Predictive mean matching was chosen from visual inspection of the data. Lasso: least absolute shrinkage and selection operator; Pmm: predictive mean matching; cart: classification and regression trees.


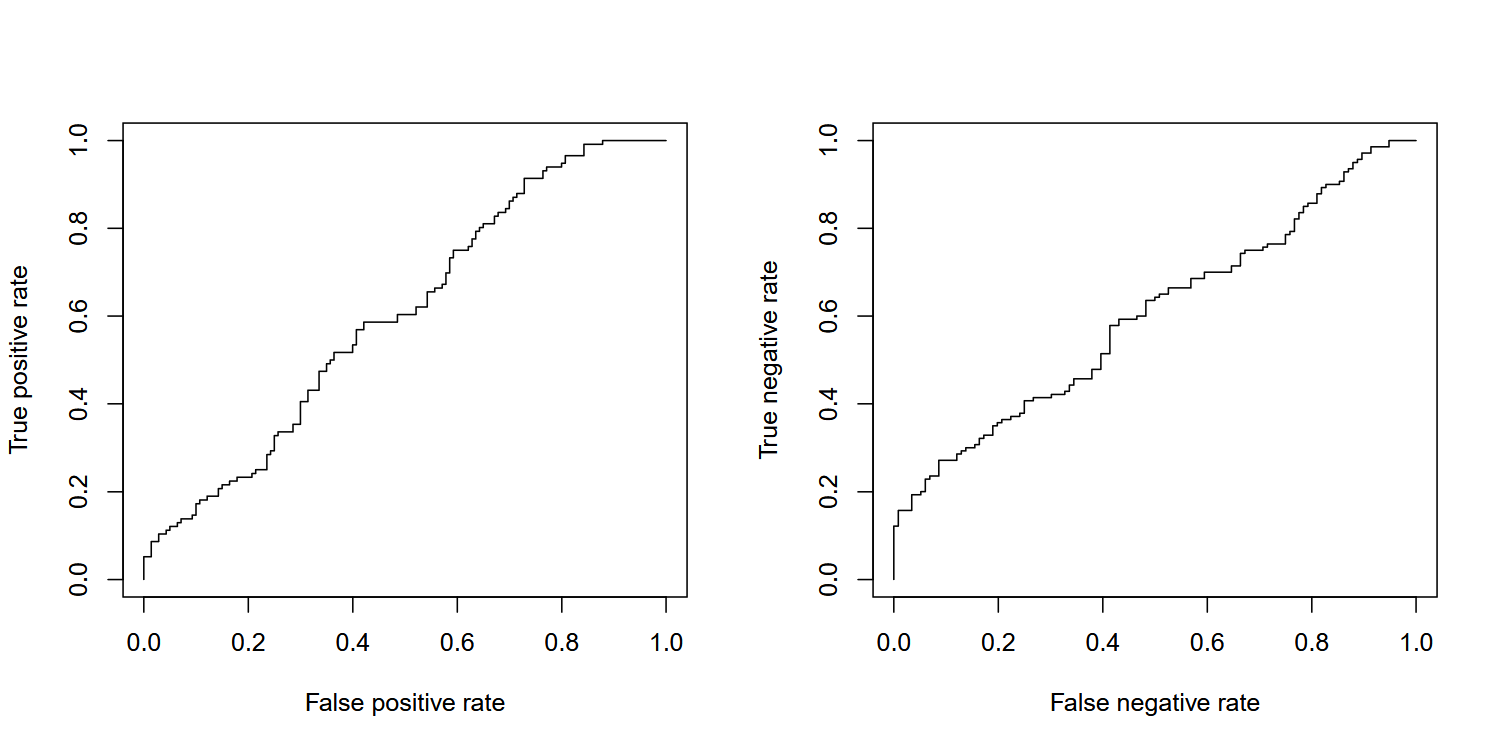


B
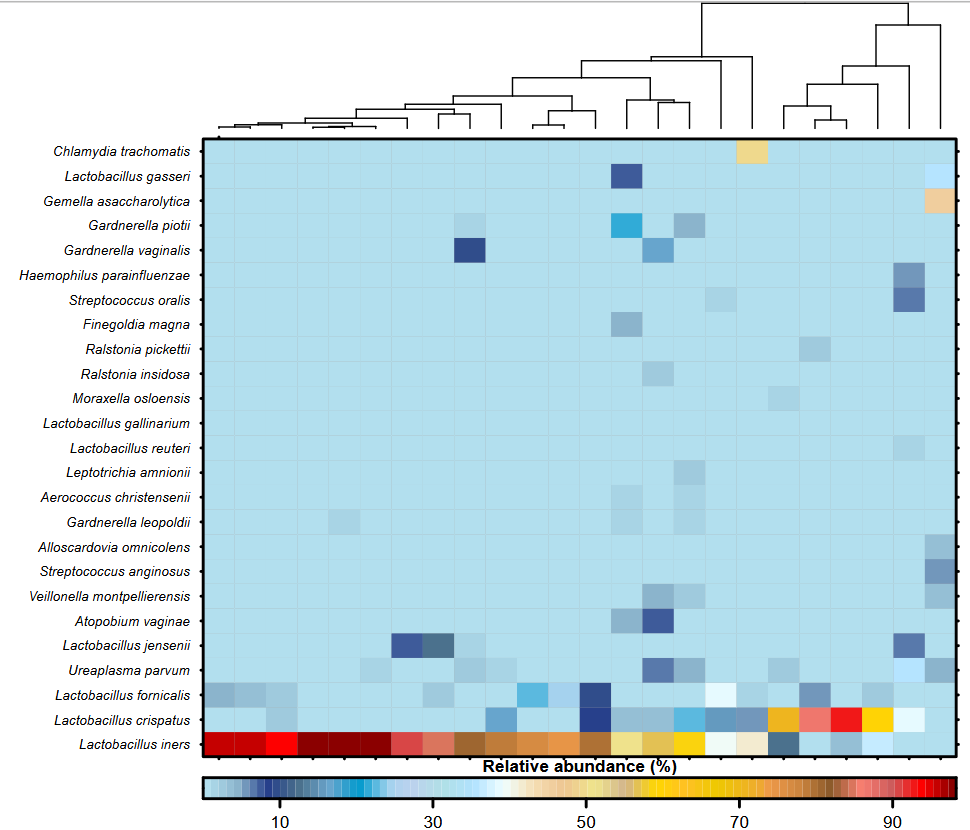


A
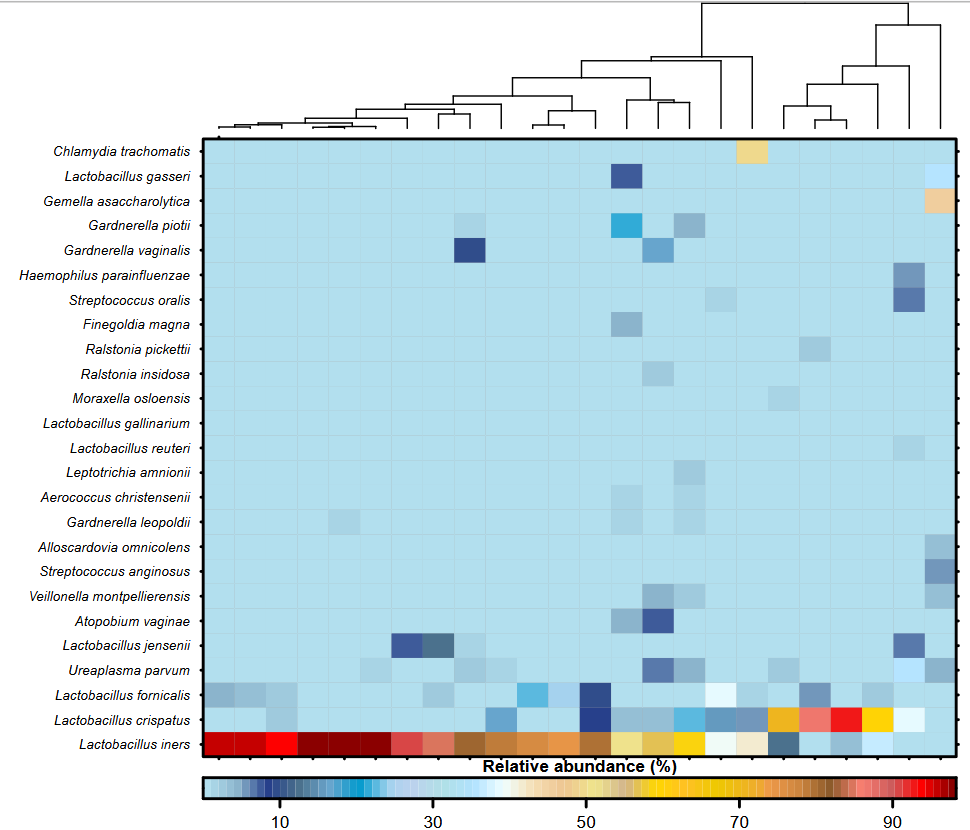


**Supplementary Figure S6: Receiver operating characteristics curves of the prediction model.** The overall predictive power of the model is limited with accuracy of 60.2%. True vs. false positive (A) and true vs. false negative (B) predictions are given for the outcome variable “embryo implantation”.

**Supplementary Materials and Methods**

**Power analysis determining the required samples size for this study**

**χ² tests -** Goodness-of-fit tests: Contingency tables

**Analysis:** A priori: Compute required sample size

**Input:** Effect size w = 0.3

α err prob = 0.05

Power (1-β err prob) = 0.95

Df = 7

**Output:** Noncentrality parameter λ = 21.8700000

Critical χ² = 14.0671404

Total sample size = 243

Actual power = 0.9503250

Central to previous findings was a difference in implantation success between the CSTs of vaginal microbiota. We, thus, calculated the requerid samples size as follows: We took the identified number if CSTs in the microbiota data set as a basis. We then calculated a Chi-square goodness-of-fit model in g*power ver. 3.1.9.7 to determine the number of samples required to identify differences in the proportion of embryo implantation between the 7 CST categories. We calculated an a priori test to detemine requeried total samples size using a medium effect size (effect size=0.3, a standard alpha (alpha=0.05) and a high power (power=0.95). The required samples size based on this calculation is 243.

**mothur script**

**Vaginal microbial community state types fail to predict IVF outcomes, whereas *Ureaplasma parvum* and *Lactobacillus iners* are negative predictors of implantation, clinical pregnancy, and live birth**

Windows version

Using Boost

mothur v.1.44.1

Last updated: 4/15/20

by

Patrick D. Schloss

Department of Microbiology & Immunology

University of Michigan

http://www.mothur.org

When using, please cite:

Schloss, P.D., et al., Introducing mothur: Open-source, platform-independent, community-supported software for describing and comparing microbial communities. Appl Environ Microbiol, 2009. 75(23):7537-41.

Distributed under the GNU General Public License

Type 'help()' for information on the commands that are available

For questions and analysis support, please visit our forum at https://forum.mothur.org

Type 'quit()' to exit program

[NOTE]: Setting random seed to 19760620.

Interactive Mode

make.file(inputdir = ., type = gz, prefix = *)

make.contigs(file = *, processors = 8)

count.groups(count = *)

summary.seqs(fasta = *, processors = *)

screen.seqs(fasta = *, group = *, maxambig = 0, maxlength = 500, processors = *)

unique.seqs(fasta = *)

count.seqs(name = *, group = *)

summary.seqs(count = *)

align.seqs(fasta = *, reference = ezbiocloud_full_align.fasta, processors = *)

summary.seqs(fasta = *, count = *, processors = *)

screen.seqs(fasta = *, count = *, summary = *, start = 9238, end = 15352, maxhomop = 12, processors = *)

summary.seqs(fasta = *, count = *)

filter.seqs(fasta = *, vertical = T, trump = .)

unique.seqs(fasta = *, count = *)

pre.cluster(fasta = *, count = *, diffs = 4, processors = *)

summary.seqs(fasta = *, count = *)

chimera.vsearch(fasta = *, count = *, dereplicate = T, processors = *)

remove.seqs(fasta = *, accnos = *, dups = F)

summary.seqs(fasta = *, count = *)

classify.seqs(fasta = *, count = *, reference = ezbiocloud_full_align.fasta, taxonomy = ezbiocloud_id_taxonomy.tax, cutoff = 80, method = knn, numwanted = 1, processors = *)

remove.lineage(fasta = *, count = *, taxonomy = *, taxon = Chloroplast-Mitochondria-unknown-Archaea-Eukaryota)

count.groups(count = *)

sub.sample(fasta = *, count = *, taxonomy = *, persample = T, size = 6800)

phylotype(taxonomy = *)

make.shared(list = *, count = *, label = 1)

classify.otu(list = *, count*, taxonomy = *, label = 1)

dist.seqs(fasta = *, cutoff = 0.10)

cluster(fasta = *, count = *, method = dgc)

make.shared(list = *, count = *, label = 0.03)

classify.otu(list = *, count = *, taxonomy = *, label = 0.03)

make.lefse(shared = *, constaxonomy = *, design = *)

**note:** original file names and enabled processors have been replaced with asterisks
